# A multi-breed GWAS for morphometric traits in four Beninese indigenous cattle breeds reveals loci associated with conformation, carcass and adaptive traits

**DOI:** 10.1186/s12864-020-07170-0

**Published:** 2020-11-11

**Authors:** Sèyi Fridaïus Ulrich Vanvanhossou, Carsten Scheper, Luc Hippolyte Dossa, Tong Yin, Kerstin Brügemann, Sven König

**Affiliations:** 1 Institute of Animal Breeding and Genetics, Justus-Liebig-University Gießen, Gießen, Germany; 2 School of Science and Technics of Animal Production, Faculty of Agricultural Sciences, University of Abomey-Calavi, Cotonou, Benin

**Keywords:** Endangered cattle breeds, Morphometric traits, Multi-breed GWAS, SNP-based genetic parameters, Functional annotations, Potential candidate genes

## Abstract

**Background:**

Specific adaptive features including disease resistance and growth abilities in harsh environments are attributed to indigenous cattle breeds of Benin, but these breeds are endangered due to crossbreeding. So far, there is a lack of systematic trait recording, being the basis for breed characterizations, and for structured breeding program designs aiming on conservation. Bridging this gap, own phenotyping for morphological traits considered measurements for height at withers (HAW), sacrum height (SH), heart girth (HG), hip width (HW), body length (BL) and ear length (EL), including 449 cattle from the four indigenous Benin breeds Lagune, Somba, Borgou and Pabli. In order to utilize recent genomic tools for breed characterizations and genetic evaluations, phenotypes for novel traits were merged with high-density SNP marker data. Multi-breed genetic parameter estimations and genome-wide association studies (GWAS) for the six morphometric traits were carried out. Continuatively, we aimed on inferring genomic regions and functional loci potentially associated with conformation, carcass and adaptive traits.

**Results:**

SNP-based heritability estimates for the morphometric traits ranged between 0.46 ± 0.14 (HG) and 0.74 ± 0.13 (HW). Phenotypic and genetic correlations ranged from 0.25 ± 0.05 (HW-BL) to 0.89 ± 0.01 (HAW-SH), and from 0.14 ± 0.10 (HW-BL) to 0.85 ± 0.02 (HAW-SH), respectively. Three genome-wide and 25 chromosome-wide significant SNP positioned on different chromosomes were detected, located in very close chromosomal distance (±25 kb) to 15 genes (or located within the genes). The genes *PIK3R6* and *PIK3R1* showed direct functional associations with height and body size. We inferred the potential candidate genes *VEPH1, CNTNAP5, GYPC* for conformation, growth and carcass traits including body weight and body fat deposition. According to their functional annotations, detected potential candidate genes were associated with stress or immune response (genes *PTAFR, PBRM1, ADAMTS12)* and with feed efficiency (genes *MEGF11 SLC16A4, CCDC117*)*.*

**Conclusions:**

Accurate measurements contributed to large SNP heritabilities for some morphological traits, even for a small mixed-breed sample size. Multi-breed GWAS detected different loci associated with conformation or carcass traits. The identified potential candidate genes for immune response or feed efficiency indicators reflect the evolutionary development and adaptability features of the breeds.

**Supplementary Information:**

**Supplementary information** accompanies this paper at 10.1186/s12864-020-07170-0.

## Background

In Sub-Saharan Africa, the cattle breeding sector comprises smallholder subsistence farms in extensive production systems, and only a few exclusively market-oriented productions. In consequence, there is a substantial gap with regard to organized breeding programs and routine performance testing, technical support and precise breeding policies. Against such a background, applying advanced breeding technologies combined with enhanced genomic statistical methods in order to conserve animal genetic resources remains a major challenge, as observed in many developing countries [1, 2]. First approaches to utilize genomic information in animal breeding in Africa were recently made, but based on limited resources [2, 3]. Nevertheless, the African continent is well-endowed with hundreds of indigenous, often endangered, animal genetic resources. In addition to being the source of livelihood for millions of poor farmers, these genetic resources represent specific socio-economic values, and they are unique for traits indicating adaptation to harsh environments [4–8].

High-throughput genotyping and genomic methods provide new opportunities for the genetic characterization and genetic management of African indigenous animal breeds. An elementary genetic and phenotypic evaluation is the prerequisite for future performance improvements. Such efforts might be the key to successful management and conservation of animal genetic resources in the light of policy developments, climate change, and diversifying market demands [9–11]. In this regard, studies addressing selection signatures analyses for heat tolerance, thermoregulation, tick resistance and trypanotolerance in African cattle breeds reflect the unique potential and advantage they possess [12–15]. Therefore, more efforts are needed to investigate the genetic architecture of functional and performance traits in native African breeds, in order to unravel their potential for future breeding development.

A great variety of study designs and methods for GWAS as established in recent years are powerful tools to study the genomic architecture of both qualitative and quantitative traits. Consequently, series of GWAS have been performed for performance and functional traits in various livestock species and breeds in European, American and Asian countries, e.g. [16–19]. In Africa, however, only a few GWAS in livestock have been reported to date [20–22], due to the difficulties in collecting valid phenotypic data in the smallholder production system, as well as the lack of resources and technologies for genotyping [23].

Three main factors are decisive to design a reliable GWAS: i) accurate phenotype and genotype data, ii) sufficient sample selection and sample size, and iii) application of adequate statistical methods [24]. With regard to further challenges such as population stratification, environmental influences and the complexity of quantitative traits, statistical methods are gradually enhanced [25, 26]. The broad availability of open-source software packages implementing innovative methods such as PLINK [27] and GCTA [28] for genome-wide complex trait analyses, in combination with continuously declining genotyping costs, open the potential for pilot GWAS in unstudied and undeveloped breeds for novel traits. Nevertheless, the recording of accurate phenotypes remains one big challenge in the African livestock-breeding context. The Food and Agriculture Organization of the United Nations (FAO) guidelines highlight morphometric traits as a good starting point to initially characterize unstudied breeds phenotypically and genetically [29]. Actually, morphometric traits comprise linear measurements of animal body sizes. Morphometric traits are routinely measurable even at early ages, and are proper early indicators for animal growth, health, welfare, and longevity [30–32]. Moreover, because of the accurate measurements (mostly in cm), morphometric traits provide an objective and better assessment of body traits than subjectively conformation traits scoring [33–35].

Several GWAS associated single nucleotide polymorphism (SNP) and genes to cattle morphometric or conformation traits. Major and commonly investigated cattle body traits for GWAS were hip height (stature), height at withers, body length, hip (rump) width, chest width, and scrotal circumference [36–38]. The heritabilities generally reported for these traits were moderate to large [39–41]. Moreover, despite the fact that the genomic architecture of cattle body traits is highly polygenic, many similarities with other livestock species, human and mammals in general, were observed [42, 43]. The identified regions were mainly involved in biological functions such as regulation of fetal growth, skeletal development, regulation of cell cycle or cell division, homeostasis, and lipid metabolism [40–44]. More interestingly, different studies identified significant overlaps in genomic architecture, and in genomic relationships between morphometric or conformation traits with cattle performance traits including body weight, carcass trait, feed intake, reproduction and health [42–46]. Indeed, phenotypic correlations between morphometric traits and animal performances such as body weight and milk offtake were very similar in various African breeds [47–49]. Furthermore, models to predict body weight from heart girth, body length or height at withers have been established [47, 48], suggesting morphometric traits as major predictors of animal performances in African livestock herds, where recording systems are poorly developed. Addressing principles of selection, Kabi et al. [50] indicated that morphometric population diversity is a result of selection for adaptive and sociocultural interests in African smallholder livestock production context. Hence, investigating genomic regions associated with morphometric traits is worthwhile in African breeds, as it allows a better understanding of animal diversity and adaptation features. In addition, its opens prospects for the effective use of morphometric traits in basic phenotype recording for any potential community-based breeding program in African smallholder livestock systems.

As discussed by many scientists [24, 51], GWAS are exploratory in their nature and further investigations based on SNP marker effects are required to get more insights into the genetic and biological basis of a trait. Hou and Zhao [52] reviewed tools and genomic features such as differential gene expressions, protein deleteriousness predictions and DNase I hypersensitive sites that could be used in understanding biological causal mechanisms and the functional relevance of identified significant SNP. In the context of limited resources, approaches that rely on documented information and public databases, such as candidate gene functional annotation and enrichment analyses of gene ontology (GO), even though non-analytical, offer new prospects for a deeper interpretation of results from GWAS [53].

Cattle in Benin are commonly kept in small herds under extensive production conditions. The indigenous cattle breeds of Benin consist of two taurine (Somba and Lagune) and two hybrid (taurine x indicine, i.e., Borgou and Pabli) breeds. These breeds were described for their adaptive potential to disease and harsh environmental conditions as well as for their importance in the livelihoods of poor farmers [5, 14, 54, 55]. However, due to their low productivity, they are increasingly threatened by indiscriminate crossbreeding with zebu animals [5, 56]. In addition, routine performance recordings and structured breeding programs have not been developed yet. The existing threats were confirmed in a genetic diversity approach considering indigenous cattle breeds from Benin, focusing on the effects of transboundary transhumance [57]. The present study builds on the dataset established in Scheper et al. [57], and combines 50 k SNP data with a basic phenotypic characterization according to FAO guidelines [29]. Given the threats surrounding the indigenous cattle breeds in Benin and in Sub-Saharan Africa in general, genetic evaluations based on marker data are an important first step to develop sustainable conservation and breeding strategies.

The aim of the present study was to estimate genetic parameters and to perform genome-wide associations for morphometric traits in four indigenous cattle breeds from different agro-ecological zones (AEZ) of Benin using medium density SNP chip data. In addition, functional annotation and gene enrichment analyses were applied to identify genes and functional loci potentially associated with morphometric traits. Finally, the indigenous Benin breeds from smallholder farms were contrasted genomically with other African livestock and exotic breeds or crossbreeds raised under improved management conditions in research stations [58, 59].

## Results

### Heritabilites, phenotypic and genetic correlations

SNP-based heritability estimates for the morphometric traits ranged between 0.46 ± 0.14 (HG) and 0.74 ± 0.13 (HW, EL, Table 1). Heart girth showed the largest genetic correlations (r_g_) with all other morphometric traits (0.38–0.80). Overall, genetic correlations among all morphometric traits ranged from 0.14 (HW with BL) to 0.85 (HAW with SH). Most of the estimated genetic correlations had small standard errors below 0.11. The largest standard error (SE = 0.28) was estimated for the genetic correlation between HG and HAW. In general, HG had larger SE for genetic correlations and for the heritability in comparison to all other traits. The phenotypic correlations (r_p_) ranged between 0.25 *±* 0.05 (HW with BL) and 0.89 ± 0.01 (HAW with SH). Heart girth and hip width were phenotypically and genetically highly correlated (r_p_ = 0.62, r_g_ = 0.72). In contrast, phenotypic and genetic correlations between EL and BL (r_p_ = 0.33, r_g_ = 0.25) were considerably lower. In addition, BL showed the smallest phenotypic and genetic correlations with other traits, especially with HW (r_g_ = 0.14–0.38, r_p_ = 0.25–0.45).

**Table 1:** Estimated phenotypic and genetic correlations among morphometric traits and their heritability. Heritabilities (in bold) are on the diagonal, above the diagonal are the genetic correlations and below the diagonal are the phenotypic correlations

	HAW	SH	HG	HW	BL	EL
HAW	**0.72 ± 0.08**	0.85 ± 0.02	0.80 ± 0.28	0.46 ± 0.05	0.25 ± 0.08	0.42 ± 0.11
SH	0.89 ± 0.01	**0.70 ± 0.08**	na	0.46 ± 0.05	0.19 ± 0.09	0.42 ± 0.13
HG	0.62 ± 0.03	0.59 ± 0.03	**0.42 ± 0.14**	0.72 ± 0.15	0.38 ± 0.15	0.58 ± 0.25
HW	0.50 ± 0.04	0.49 ± 0.04	0.58 ± 0.03	**0.74 ± 0.13**	0.14 ± 0.10	0.35 ± 0.17
BL	0.37 ± 0.04	0.32 ± 0.04	0.45 ± 0.04	0.25 ± 0.05	0**.73 ± 0.10**	0.25 ± 0.10
EL	0.35 ± 0.04	0.33 ± 0.04	0.29 ± 0.04	0.25 ± 0.05	0.33 ± 0.04	**0.74 ± 0.10**

*HAW* height at withers, *SH* sacrum height, *HG* heart girth, *HW* hip width, *BL* body length and *EL* ear length

### Multi-breed GWAS for morphometric traits and functional annotation of candidate genes

**Fig. 1:**
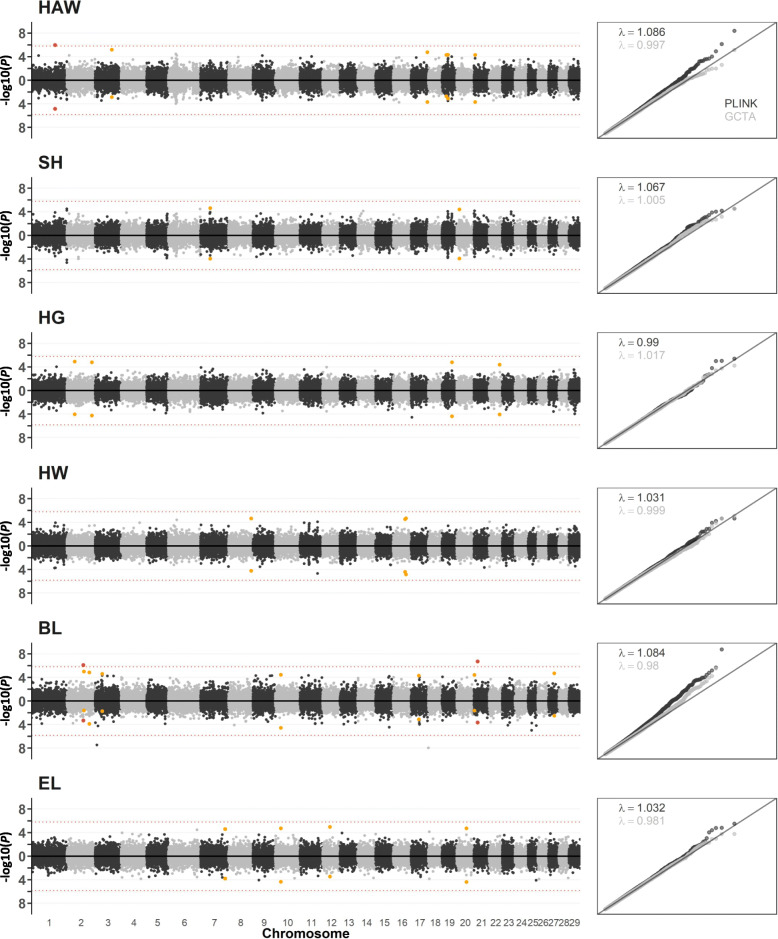
Manhattan Plots and QQ-plots displaying GWAS results from PLINK (above the x-axis) and GCTA (below the x-axis) for six morphometric traits in four indigenous cattle breeds from Benin. The genome-wide significant SNP and chromosome-wide significant SNP are displayed in red and yellow, respectively. HAW = height at withers, SH = sacrum height, HG = heart girth, HW = hip width, BL = body length and EL = ear length

Discriminant analysis of principal components (DAPC) identified four linear discriminant functions (LDF) validly representing the genetic structure in the sampled population (Additional file 1, Figure S1). The inclusion of the LDF in the PLINK GWAS resulted in sufficient correction of population stratification with desired lambda values (λ = 0.99–1.09). Slightly lower lambda values (λ = 0.98–1.00) were obtained from GWAS using GCTA (Fig. 1). The GWAS via PLINK detected a total of 28 SNP for all six morphometric traits, and the majority of these SNP were also detected via GCTA (Fig. 1). The significant SNP from PLINK were positioned within or near 15 different genes (Table 2).

**Table 2:** Genome-wide and chromosome-wide significant SNP and potential candidate genes associated with six morphometric traits from four indigenous breeds in Benin

*SNP_rs*	SNP	CHR	BP_ARS1.2	Alleles		MAF	BETA		*P*-value		SNP in Gene	Gene Name
				A1	A2		PLINK	GCTA	PLINK	GCTA		
Height at withers												
*rs109126926*^*a*^	ARS-BFGL-NGS-57889	1	110,160,486	A	G	0.26	2.24	2.16	1.12e-06	1.44e-05	Yes	*VEPH1*
*rs43347748*	ARS-BFGL-NGS-31952	3	81,139,242	C	T	0.10	2.60	1.96	6.44e-06	1.40e-03	–	*–*
*rs41634361*	Hapmap50686-BTA-41836	17	68,037,062	G	A	0.10	2.58	2.40	1.71e-05	1.99e-04	Yes	*CCDC117*
*rs110369628*	ARS-BFGL-NGS-91812	19	20,892,297	T	C	0.02	5.58	4.55	4.82e-05	2.00e-03	Yes	*SSH2*
*rs109889052*	ARS-BFGL-NGS-81151	19	28,301,046	A	G	0.13	2.35	2.08	4.57e-05	7.94e-04	Yes	*PIK3R6*
*rs109872376*	ARS-BFGL-NGS-46597	21	7,674,101	C	G	0.44	1.62	1.54	5.04e-05	2.09e-04	–	*–*
Sacrum height												
*rs111001850*	ARS-BFGL-NGS-7310	7	42,545,291	A	G	0.45	1.53	1.50	2.35e-05	1.12e-04	No	*LYPD8*
*rs110441360*	ARS-BFGL-NGS-110086	20	11,381,378	A	G	0.48	−1.51	−1.51	3.86e-05	1.19e-04	No	*PIK3R1*
Heart girth												
*rs110404606*	ARS-BFGL-NGS-77689	2	32,163,209	C	T	0.38	2.83	2.55	1.17e-05	9.65e-05	–	*–*
*rs41579167*	BTA-49621-no-rs	2	125,244,296	G	A	0.44	−2.93	−2.75	1.55e-05	5.59e-05	No	*PTAFR* *EYA3*
*rs41624005*	Hapmap48676-BTA-18047	19	46,729,603	A	G	0.50	2.72	2.64	1.60e-05	4.29e-05	–	*–*
*rs41637645*	Hapmap39844-BTA-54797	22	48,208,654	C	A	0.16	−3.48	−3.36	4.10e-05	8.96e-05	Yes	*PBRM1*
Hip width												
*rs109866742*	ARS-BFGL-NGS-119529	8	103,907,956	T	C	0.02	−3.12	−3.06	2.11e-05	5.89e-05	–	*–*
*rs41569598*	BTA-39611-no-rs	16	56,426,595	C	T	0.43	−0.97	−0.97	2.99e-05	3.88e-05	–	*–*
*rs42843320*	BTB-01732320	16	60,525,984	C	T	0.22	−1.12	−1.15	2.08e-05	1.47e-05	Yes	*ABL2*
Body length												
*rs110694334*^*a*^	ARS-BFGL-NGS-109828	2	76,610,609	C	T	0.03	−17.96	−11.94	8.19e-07	4.90e-04	yes	*CNTNAP5*
*rs109186122*	ARS-BFGL-NGS-118432	2	78,925,610	C	T	0.25	−7.18	−3.43	9.33e-06	2.48e-02	yes	*GYPC*
*rs42301516*	BTB-01145402	2	113,155,761	G	A	0.43	−5.45	−4.31	1.38e-05	1.37e-04	–	*–*
*rs135705191*	BovineHD0300010335	3	33,048,892	G	A	0.20	−6.85	−3.72	2.49e-05	1.90e-02	yes	*SLC16A4*
*rs43616983*	BTB-00409355	10	12,969,418	G	A	0.20	6.70	6.20	3.41e-05	2.94e-05	yes	*MEGF11*
*rs42436268*	BTB-01308172	17	33,487,976	G	T	0.32	5.45	4.06	4.93e-05	8.18e-04	–	*–*
*rs41608167*	BTA-96370-no-rs	21	3,408,269	T	C	0.02	−18.36	−9.53	3.74e-05	2.29e-02	–	*–*
*rs41607390*^*a*^	Hapmap33092-BTA-51753	21	18,775,375	T	C	0.11	−10.39	−7.07	2.03e-07	2.20e-04	–	*–*
*rs41646754*	Hapmap44720-BTA-62525	27	24,546,315	G	C	0.27	−6.28	−4.04	1.91e-05	3.27e-03	–	*–*
Ear length												
*rs109212458*	ARS-BFGL-NGS-85383	7	96,266,192	T	C	0.33	0.42	0.38	2.54e-05	1.63e-04	–	–
*rs110608572*	ARS-BFGL-NGS-103122	10	12,919,428	A	G	0.03	1.10	1.07	1.86e-05	4.40e-05	Yes	MEGF11
*rs41567897*	BTA-100327-no-rs	12	41,375,572	C	G	0.17	−0.59	−0.49	1.08e-05	3.56e-04	–	–
*rs109985119*	ARS-BFGL-NGS-44763	20	40,060,386	T	C	0.14	0.56	0.54	1.90e-05	4.23e-05	Yes	ADAMTS12

^a^Genome-wide significant SNP; the remaining SNP are the chromosome-wide significant SNP

The significant SNP, potential candidate genes and their functional annotation are presented in the ongoing sub-chapters for the morphometric traits, considering two traits per sub-chapter.

#### Height at withers and sacrum height

Height at withers was significantly associated with the SNP *rs109126926* on BTA1 (*p =* 1.12e-06). In addition, five chromosome-wide suggestively associated SNP were detected on chromosomes 3, 17, 19 and 21 (*p* = 6.44e-06 - 5.04e-05); see Table 2 for the exact positions). The SNP *rs110369628* and *rs109889052* on BTA19 were positioned in relative proximity to each other (7.41 Mb), within the *PIK3R6* and *SSH2* genes, respectively. The *VEPH1* gene harbored the significantly associated SNP for HAW on BTA1, while another SNP, *rs4163436*, was positioned in *CCDC117* on BTA17*.* No genes were annotated for the two remaining significantly associated SNP*.*

Only two SNP were suggestively associated with SH (*p* = 2.35e-05 - 3.86e-05) and each was located in close distance to a gene. The SNP *rs111001850* was positionally linked to the *LYPD8* gene on BTA7, and the SNP *rs110441360* was located near the *PIK3R1* gene on BTA20.

#### Heart girth and hip width

No SNP surpassed the genome-wide significance threshold for associations with HG and HW. However, four SNP were suggestively associated with HG (*p* = 1.17e-05 - 4.10e-05). Two of them were positioned on BTA2 and the two others were located on BTA19 and BTA22. The SNP *rs41579167* on BTA2 was positioned near the two genes *PTAFR* and *EYA3*. The gene *PBRM1* harbored the SNP *rs41637645* on BTA22.

Hip width was associated with three suggestive SNP (*p =* 2.08e-05 - 2.99e-05). Two of the three SNP were positioned relatively near to each other (4 Mb) on BTA16. The third SNP was detected on BTA8. Only the *ABL2* gene was mapped as a potential candidate gene for HW, harboring the SNP *rs42843320* on BTA16.

#### Body length and ear length

Body length presented the highest number of associated SNP with two significant and 7 suggestive SNP. BTA2 harbored one significant SNP (*rs110694334, p =* 8.19e-07*)* along with two others suggestive SNP (*p* = 9.33e-06 - 1.38e-05). Similarly, BTA21 harbored the second significant SNP (*rs41607390, p =* 2.03e-07) and one suggestive SNP (*p* = 3.74e-05), but none of them was positionally linked to a gene. The other associated SNP variants were identified on chromosomes 3, 10, 17 and 27 (*p* = 1.91e-05 - 4.93e-05). On BTA2, the SNP *rs110694334* was positioned within the *CNTNAP5* gene, in relative proximity (2.32 Mb) to the SNP *rs109186122* positioned in the *GYPC* gene*.* Two additional genes, *SLC16A4* and *MEGF11*, were mapped as potential candidates for BL, harboring the SNPs *rs135705191* and *rs43616983* on BTA3 and BTA10, respectively.

Four SNP positioned on different chromosomes (7, 10, 12, 20) were suggestively associated with EL (*p* = 1.08e-05 - 2.54e-05). The SNP *rs110608572* SNP was positioned on BTA10 within the *MEGF11* gene*,* which was also identified as a potential candidate gene for BL. The SNP *rs109985119* was located in the *ADAMTS12* gene on BTA20.

## Discussion

### Heritabilities, phenotypic and genetic correlations

The heritability estimates for morphometric traits obtained in the present study were larger than those commonly reported for comparable linear body traits [39–41, 60–63]. However, heritability estimates are usually higher for morphometric traits (measurements) [39–41] than for conformation traits (scores) [60, 61, 63]. Hence, quite large heritability estimates (up to 0.7) have been similarly reported in few studies based on morphometric traits [64–67]. For instance, the heritability estimate of 0.42 for HG in our study is comparable to the value of 0.43 reported for adult Brahman cattle [66]. Moreover, the heritabilities of 0.72 for HAW and of 0.70 for SH are in agreement with the estimates for stature in Brahman (0.73) [65] and in Red & White cattle (0.74) [64]. These observations suggest that real and objective measurements provide a better basis for heritability estimations than subjective conformation scores.

The estimates of genomic correlations for the different trait combinations ranged from 0.14 to 0.84, with standard errors between 0.02 to 0.25. Roveglia et al. [63] reported a broad range (0.08 to 0.98) for genetic correlations among conformation traits reflecting the morphometric characteristics from the present study. The genetic correlation between HW and height traits (0.46) in the present study was smaller than the estimate of 0.75 [63]. Nevertheless, generally lower genetic correlations (0.09 to 0.35) between these trait combinations have been previously described [64, 68]. The genetic correlation of 0.80 between HG and HAW from the present study is larger than the values that were previously reported (0.30–0.65) [40, 64, 69, 70]. The large genetic correlations between HG with the other morphometric traits are in agreement with results from a previous study [64].

The estimates of phenotypic correlations between height traits (HAW and SH) and HG (0.59 and 0.62) were slightly larger than those (0.20 to 0.50) reported in previous studies [40, 70]. However, regarding the comparisons made, it is imperative to state that our study is the only one using genomic instead of pedigree relationships. To our knowledge, this is the first study, which estimated phenotypic and genetic correlations among morphometric or conformation traits on the basis of SNP marker data. Moreover, the cited genetic parameters estimates used for the comparisons are from Holstein, Brahman, Brown Swiss and Red & White populations kept in Europe or Asia [40, 63–66]. The few genetic evaluations in the African context were made for growth traits (birth, weaning and yearling weight) in exotic breeds kept under controlled management conditions in research stations [58, 59]. In African smallholder farms, genetic parameters only have been estimated for milk production, considering exotic breeds and crossbreeds (exotic × indigenous) [71].

As a major difference to previous studies, our approach focused on multi-breed genetic parameter estimations. Meyer et al. [72] observed larger heritabilities and genetic variances in multi-breed populations, compared with estimates in purebred populations. These findings are in agreement with the moderate to high values for genetic parameters obtained in our study. However, high genetic variability also was identified in single breeds, but potentially be biased due to correlations between environments and genotypes, and due to extremely close genetic relationships [73]. Thus, accounting for population structure particularities remains a great challenge in all studies estimating genetic parameters, even within a single breed [73]. Our study attempted to avoid relatedness in the dataset by sampling animals from different herds and AEZ. Nevertheless, breed admixture was oberved between and within the indigenous breeds in Benin, due to continuous indicine introgression and uncontrolled crossbreeding [57]. Thus, breed specific modelling approaches might be affected from within-breed diversity. Overall, the heritabilities, phenotypic and genetic correlations from the present multi-breed study reflect the range for single-breed estimates, indicating that most of the genetic variations in body traits is based on universal common rather than on breed specific genetic variants [74]. Indeed, multi-breed population references enhance the potential in detecting common and conservative loci [72, 75]. In this regard, Wientjes et al. [76] focused on model comparison, and observed most accurate genetic parameters from models capturing large proportions of the genetic variance in a population. Therefore, in the context of uncontrolled crossbreeding as prevalent in indigenous cattle breeds in Africa, a multi-breed approach might be useful.

### Multi-breed GWAS for conformation traits and functional annotation of candidate genes

#### Height at withers and sacrum height

For the SNP *rs109126926* SNP significantly associated with HAW and the corresponding gene *VEPH1*, no direct association between the variant or the identified gene with cattle height were previously reported. However, *rs109126926* was significantly associated with recoverability from mastitis in Holstein cows [77]. *VEPH1* influenced other cattle conformation traits such as udder cleft in Holstein and rump fat thickness in Nellore cattle, reflecting its role in lipid metabolism [78, 79]*.* Furthermore, recent studies observed an association of *VEPH1* with residual feed intake and antibody response to parasites in cattle [80, 81].

Among the five suggestive SNP associated with HAW, *rs109889052* and *rs110369628* were located on BTA19. These two SNP have not been directly associated with cattle height, but *rs109889052* contributed to feed efficiency in cattle [82]. Moreover, *rs109889052* is located in the *PIK3R6* gene, affecting body size in sheep [31]. Likewise, *rs110369628* positioned in the *SSH2* gene, was linked to carcass traits in sheep [83]. According to the database for annotation, visualization and integrated discovery (DAVID), *SSH2* is involved in the regulation of the actin cytoskeleton pathway and in several other molecular and biological functions such as the regulation of actin polymerization or depolymerization and DNA binding. In previous studies, *SSH2* was associated with somatic cell score and semen traits in cattle [84, 85]. Bouwman et al. [42] identified several SNP on BTA19 associated with cattle stature, including *rs132693733* (19:21339030), *rs109018020* (19:23816722) and *rs137732346* (19:25980624). These markers are positioned in a distance between 0.45 Mb and 6.96 Mb from the SNP identified in our study, i.e., *rs109889052* (19:20892297) and *rs110369628* (19:28301046). Another variant *rs42741630 (*19:25439551) in close proximity (2.86 Mb) to *rs110369628* (19:28301046), was detected in a recent GWAS for stature [86]. These findings suggest that this chromosomal segment on region BTA19 (at 20 Mb to 30 Mb) might represent a hotspot genomic region for height. We found no reference for the three other variants associated with HAW in the literature. Nevertheless, the SNP *rs41634361* on BTA17 is positioned within the *CCDC117* gene*,* which is involved in feed intake and heat stress regulation in cattle [87, 88].

The two SNP suggestively associated with SH were not associated with cattle height in previous studies. However, the SNP *rs110441360* on BTA20 is positioned in the *PIK3R1* gene, which influenced beef fat content [89]. More interestingly, different studies described *PIK3R1* for its implication in the human short stature syndrome [90, 91], confirming the functional conservation of genes linked to stature or body size in cattle and humans [42–44]. Moreover, these findings are consistent with the functional annotation of *PIK3R1* in protein stabilization, insulin resistance, and growth hormone receptor signaling pathways. The potential effect of growth hormone receptor genes on animal conformation traits has been previously reported [92]. The second variant (*rs111001850)* associated with SH is located in the *LYPD8* gene*.* This gene is involved in biological processes of defense responses to gram-negative bacteria, and has been related to adaptive responses to environmental stimuli such as stress, infection and inflammation in cattle [93].

The association of two PIK regulatory subunit genes with height traits in this study is remarkable. Another *PI3K regulatory subunit* gene, i.e.*PIK3R5*, was identified in a region highly associated with body size in sheep [31]. Moreover, *PIK3R6* and *PIK3R1* are declared as potential candidates for feed intake and feed efficiency in cattle [82, 94]. Both genes are members of a metabolism pathway, specifically involved in the synthesis of PIPs at the plasma membrane. In addition, we found that *PIK3R6* and *PIK3R1* are involved in several animal immune system pathways or related biological processes, such as the B cell receptor signaling pathway, the AMPK signaling pathway and regulation of T cell differentiation. Moreover, they are members of different signal transduction pathways (G beta: gamma signaling through PI3Kgamma, signaling by SCF-KIT), which are also linked to inflammatory diseases [95, 96]. The involvement of *PIK3R6* and *PIK3R1* in signal transduction and immunity pathways reflect the associations with resistance to *Mycobacterium avium ssp. paratuberculosis* (MAP) infections in cattle [27–29].

Indigenous cattle breeds in Benin, especially the taurine breeds, are known to be resistant against diseases [14, 54, 55]. In comparison to other breeds, they are small sized, and kept in harsh environments with limited feed resources and a high disease infection risk. In this context, HAW is described as an indicator of animal adaptive attributes [29]. Hence, our findings for HAW and SH confirm that *PIK3R6* and *PIK3R1* may play an important role in the interaction of adaptation to diseases and body size variability in African cattle, and potentially in cattle populations worldwide.

#### Heart girth and hip width

The four SNP suggestively associated with HG were not detected in any previous GWAS for HG. However, two of them are positionally linked to three genes influencing dairy cattle traits. First, the SNP *rs41579167* SNP is located in direct proximity to the genes *PTAFR* and *EYA3.* These two genes are involved in inflammatory responses and both are associated with immune response in cattle [97, 98]. In addition, *PTAFR* is associated with intramuscular fat deposition in Nellore cattle [99] and was identified in selective regions for production performance in different cattle breeds [100, 101]. Furthermore, in mice, *PTAFR* affected body weight by controlling feed intake and obesity [102]. According to DAVID, the *EYA3* gene is involved in cell differentiation processes and in a pathway related to DNA repair mechanisms. This gene was also identified as a potential candidate gene in a GWAS for milk production in dairy sheep [103]. The SNP *rs41579167* SNP (2:125244296) is located at 0.15 Mb distance to another variant *rs2083797338* (2:125093797), which has been identified in a meta-GWAS for cattle stature [42].

Second, the SNP *rs41637645* is located within *PBRM1,* a gene which was associated with heat stress regulation in tropical breeds [104]. *PBRM1* is also involved in the negative regulation of cell proliferation and in the RMTs methylate histone arginine pathway, while this pathway is involved in different diseases in mammals [105].

We found no direct link between the four suggestively associated SNP for HW with HW in previous studies. Nonetheless, *ABL2* is a potential candidate gene for HW, harboring *rs42843320*. *ABL2* influenced feed intake in cattle and backfat thickness in pigs [106, 107]. In addition, *ABL2* is involved in innate immune response processes through cell proliferation, migration and differentiation (according to its functional annotation in the DAVID database).

#### Body length and ear length

Body length was significantly associated with *rs41646754* on BTA27, and with *rs110694334*, which is located within *CNTNAP5* on BTA2*.* Neither the two variants, nor the identified genes, have been previously associated with BL. Nevertheless, *CNTNAP5* was detected in a chromosomal segment significantly associated with hip cross height in Brahman cattle [108]. Furthermore, *CNTNAP5* is a potential candidate gene for conformation traits in Sudanese goats and growth traits and pigs [109–111]. In addition, *CNTNAP5* was identified in a selective region for adaptation in cattle as well as in sheep [112, 113]. According to DAVID, *CNTNAP5* is an integral component of membranes and is related to the epidermal growth factor-like protein domain.

Among the seven suggestive SNP associated with BL, *rs43616983* on BTA10 was associated with direct perinatal mortality in Holstein–Friesian dairy cattle [16]. Moreover, *rs43616983* is positioned within the *MEGF11* gene*,* which regulated daily gain and immune response to mastitis in cattle [85, 114]. Another study observed different expressions of *MEGF11* in the *musculus longissimus dorsi* of two different cattle breeds in response to low energy diets [85]. This finding is consistent with results from GWAS in pigs suggesting *MEGF11* as a potential candidate gene for feed efficiency [115]. Furthermore, *MEGF11* was significantly associated with height in Buffalo [116]. Two other SNP suggestively associated with BL, *rs109186122* and *rs43616983*, are located in the genes *GYPC* and *SLC16A4*, respectively. According to its annotation in DAVID, *GYPC* is involved in oligosaccharide binding and is reported to be associated with intramuscular fat deposition and reproduction performances in cattle [117–119]. The *SLC16A4* gene had effects on postweaning weight gain, feed efficiency and resistance to the bovine viral diarrhea virus in cattle [120–122]. These observations are in line with the involvement of *SLC16A4* in transmembrane transport functions and glucose import processes, as well as with the impact of the SLC16 gene family on health regulation [123]. The *SLC16A4* gene is located in a genomic segment being under divergent selection in South African cattle breeds [13]. In addition, solute carrier family genes were associated with cattle body weight or conformation traits [124, 125].

None of the four SNP variants suggestively associated with EL are linked to EL in the literature, because of the trait relevance only for tropical production systems. Two potential candidate genes for EL are related to different traits in cattle and other mammal species. Firstly, *MEGF11*, harboring the SNP *rs110608572* on *BTA10,* overlap with our findings for BL. Secondly, *ADAMTS12,* harboring the SNP *rs109985119* on BTA20, was associated with body weight and supernumerary teat in cattle [126–128]. *ADAMTS12* was involved in inflammatory responses and in the regulation of the hepatocyte growth factor (HGF) receptor signaling pathway. Indeed, HGFs are known to play an important role in the stimulation of epithelial cell proliferation, motility, morphogenesis and angiogenesis [129]. The *ADAMTS12* gene was identified in pigs in a selection signature associated with genetic adaptation to high altitude [130]. These observations confirm a potential effect of *ADAMTS12* on EL variability, considered as an adaptive trait for heat tolerance in the indigenous breeds in Benin [29, 131].

### Genomic regions associated with morphometric and adaptation traits

The comparison with the literature gives convincing evidence for the validity of our results based on gene functions and associations as detected in other traits and species. The rather small number of detected SNP for the moderately to highly heritable polygenic traits is in line with our small sample size and its diversity (multi-breed), and is most likely due to a lack of power to detect variants with smaller effects. However, with regard to the functional annotations of the identified genomic regions, our study confirms the potential of multi-breed GWAS in detecting fewer variants, but more precise functional loci or causative mutation across breeds [75, 132]. In this context, the SNP associated with morphometric traits in the present study may represent novel common variants for linear body traits in African breeds, suggesting further investigations. Furthermore, the validity of the multi-breed approach may constitute to collaborative research towards better characterizations and genetic evaluations, including the animal genetic resources in Africa [2, 3, 71, 133].

Our findings reveal a close connection between genomic regions associated with morphometric traits and adaptive traits. For instance, two of the identified variants detected for morphometric traits in this study are directly linked to immune response, while several potential candidate genes have functional annotations for, e.g., signal transduction, metabolism, and immune response adaptation [134]. The comparison with previous studies confirms associations of the identified genes with selection signatures as well as immunity or resistance to diseases, feed efficiency and adaptation to harsh environments (heat stress, high altitude), addressing the main components of adaptation [4]. On the one hand, our observations are consistent with the breeding history of the indigenous breeds in Benin, which is characterized by natural and non-directional selection based on individual farmer preferences. In many African breeds, adaptive traits (disease resistance, feeding ease) and reproductive performances reflect the major breeding preferences of farmers, and they select animals according to their morphometric or conformation appearance rather than on actual recorded performance [50, 135, 136]. Hence, this might be an explanation for the observed association between morphometric and adaptive traits in our study [50, 137].

On the other hand, genomic regions associated with adaptive traits (immune response or feed efficiency) have been similarly detected in GWAS for linear body traits in more developed breeds such as Holstein and Angus [36, 62, 92, 138]. These overlaps as well as the effect of selection response for disease resistance or feed efficiency on conformation or carcass traits, and vice-versa, have been extensively discussed [138, 139]. In our view, two hypotheses emerge from such overlap. First, the identified loci or genomic regions may simultaneously control morphometric traits and adaptive traits, indicating the pleiotropic role of many loci associated with body conformation traits such as height or body size [42]. Secondly, a high genetic correlation between morphometric and adaptive traits is due to the long history of natural selection in the studied breeds on both trait categories.

## Conclusion

Heritabilities for as well as phenotypic and genetic correlations among morphometric traits based on dense SNP marker data and a multi-breed approach were moderate to moderately high. Twenty-five SNP and fifteen genes potentially associated with the morphometric traits were detected. Comparisons with previous studies and the functional annotation of the genes revealed a clear association of loci identified in this study with conformation, growth and carcass traits in cattle or in other species. Moreover, the majority of the detected genes are associated with immune response and feed efficiency, or involved in related biological processes, suggesting a strong correlation between morphological and adaptive traits. This is in line with the evolutionary development and breeding history in these indigenous breeds mainly shaped by natural selection. Our findings suggest that accurate phenotyping (measurements) for morphometric traits combined with SNP marker data can be used for genetic evaluations, considering mixed-breed cattle populations.

## Methods

### Phenotypes and animal resources

Following the FAO guidelines [29], we recorded six morphometric traits (Table 3) on 449 animals from the four main indigenous cattle breeds in Benin (Borgou 181, Pabli 58, Lagune 150, Somba 60). The morphometric traits were chosen for their importance in cattle characterization and their association with production or adaptive traits. Sacrum height, heart girth, hip width and body length are commonly used to evaluate cattle body size and growth, and they are highly correlated with body weight or milk yield in different cattle breeds [40, 44, 47, 48, 66, 140]. In addition, ear length and height at withers are related to adaptive traits, whereas hip width is associated with animal longevity [29, 30].

**Table 3:** Description of the six morphometric traits recorded on four indigenous cattle breeds from Benin

Morphometric traits	Description	Measuring device
Height at withers	Vertical distance from the bottom of the front foot to the highest point of the shoulder between the withers	Measuring stick
Sacrum height	Distance from the top of the bone at the base of the tail to the ground	Measuring stick
Heart girth	Circumference of the body immediately behind the shoulder blades in a vertical plane, perpendicular to the long axis of the body	Measuring tape
Hip width	Distance between the rearmost posterior points of pin bones	Wooden caliper
Body length	Horizontal distance from the point of the shoulder to the pin bone	Measuring tape
Ear length	Length on the back side of the ear from its root on the poll to the tip	Measuring tape

The animals were selected from a larger dataset of 462 animals as described in a previous study [57]. Thirteen animals from the larger dataset were excluded from the current study due to inconclusive genetic adherence and impact from crossbreeding [57]. The animals were sampled in small cattle herds kept under extensive management, according to their distribution across AEZ in Benin (see Additional file 2, Table S1 for animal characteristics, location and geographic coordinates of the herds). Somba and Pabli cattle were sampled in the AEZs Ouest Atacora (OA) and Cotonnière Nord (CNB). Borgou cattle were sampled in three different AEZs Cotonnière Centre (CCB), Vivrière Sud Borgou (VSB) and Cotonnière Nord (CNB), and the Lagune cattle in three other AEZs Pêcheries (P), Dépression (D) and Terre de Barre (TB). One-way analysis of variance (ANOVA) tests for the six morphometric traits revealed significant differences *(p ≤ 0.001)* across breeds with higher values for hybrid breeds (Borgou and Pabli). For instance for height at withers, Borgou and Pabli cattle measured 116.5 ± 5.67 cm and 111.4 ± 7.58, but Somba and Lagune are smaller with 96.37 ± 4.97 cm and 92.59 ± 7.18, respectively. The full description of the six morphometric traits for the four breeds are presented in Additional file 3, Figure S2.

### Genotypes, quality control and imputation

The 449 selected animals with phenotypes were genotyped using the Illumina BovineSNP50 BeadChip, and 51,278 SNP were available before quality control. Sample collection, DNA extraction and genotyping procedures are described in detail in Scheper et al. [57].

Genotyping quality control was performed using the PLINK software [26] to retain SNP with a minor allele frequency larger than 5% and a genotyping call rate of 90%, and which are in Hardy-Weinberg equilibrium (*p* ≥ 10^− 06^). For individual animals, a genotype call rate larger than 95% was required. After quality control, 14,518 SNP and one animal were discarded, implying a genotype dataset with 36,760 SNP variants from 448 cattle.

Sporadic missing variants were imputed with BEAGLE [141] after remapping the SNP positions to the current reference assembly ARS1.2 and removing all markers with unknown position on ARS1.2. Genotype imputation generated a dataset of 36,720 SNP for the ongoing genomic analyses.

### Adjustment for fixed and environmental effects and genetic structure

One-way ANOVA in R was firstly applied on each morphometric trait to test the explanatory variables for significance. Given that breeds are nested within AEZ, a new variable (AEZ_Breed), which combines the two variables AEZ and breed, was created. The factors age, sex, and AEZ_Breed were simultaneously included in a multi-factor linear model to test their effects on the respective morphometric trait via the Type III sums of squares from ANOVA, using the Car package in R [142]. AEZ_Breed showed significant effects on the six morphometric traits *(p ≤ 0.001)*, while sex had significant effect only on HG, HW and BL, and age on HG, HW and HAW (Additional file 4, Table S2). In consequence, AEZ_Breed, sex and age were considered as fixed effects in the GWAS models for all morphometric traits.

The genetic structure in the dataset was evaluated applying a DAPC, using the R package ADEGENET [143, 144]. LDF were used as covariates in addition to AEZ_Breed, sex and age to correct for population stratification in GWAS. DAPC was chosen instead of classical principal component analysis (PCA) as it better characterizes the genetic structure of the population [145, 146] (see Additional file 4, Table S3, for significant SNP when the first four PCs were included as covariates in the GWAS model). The genotype dataset used for DAPC consisted of 25,065 SNP after pruning of the imputed dataset based on linkage disequilibrium (LD) between markers. The *“--indep-pairwise*” command in PLINK and defining 0.2 for the r^2^ threshold, was considered in this regard [27].

### Estimation of heritability, phenotypic and genetic correlation

A genomic relationship matrix between the animals was firstly generated with the -*grm* method in GCTA, and afterwards considered for the estimation of the genetic parameters [28, 74]. The restricted maximum likelihood (REML) method was applied for genetic parameter estimation. The respective genetic-statistical model was defined as follows:

1 $$ \mathbf{y}=\mathbf{Xb}+\mathbf{Zu}+\mathbf{e} $$

where **y** was a vector of morphometric traits; **b** was a vector of fixed effects including age, sex, and AEZ_Breed; **u** was a vector of polygenic effects with a variance-covariance structure of $\mathbf{u}\sim N\left(0,\mathbf{G}{\sigma}_u^2\right)$, **G** was the genomic relationship matrix between individuals [74], ${\sigma}_u^2$ was the polygenic variance; **e** was a vector of random residual effects with $\mathbf{e}\sim N\left(0,\mathbf{I}{\sigma}_e^2\right)$, **I** was an identity matrix of dimension *n* × *n* (with *n*, the sample size = 449); and **X** and **Z** were incidence matrices for **b** and **u**, respectively.

Considering two conformation traits *x* and *y*, the genetic correlation (r_g_) between *x* and *y* was estimated using the “*--reml-bivar x y”* option in bivariate genomic REML analyses. The phenotypic correlation (r_p_) between *x* and *y* was calculated from the bivariate genomic REML outputs using the following formula:

2 $$ {r}_{p_{xy}}=\frac{\sigma_{u_{xy}}+{\sigma}_{e_{xy}}}{\sqrt{\left({\sigma}_{u_x}^2+{\sigma}_{e_x}^2\right)\times \left({\sigma}_{u_y}^2+{\sigma}_{e_y}^2\right)}} $$

where ${\sigma}_{u_{xy}}$ and ${\sigma}_{e_{xy}}$ were the genetic covariance and residual covariance between *x* and *y*, respectively; and ${\sigma}_u^2$ and ${\sigma}_e^2$ were the genetic variance and residual variance of *x* and *y*, respectively. The standard errors of the phenotypic correlation were calculated based on the “deltamethod” function from the R-package “msm” [147].

### Multi-breed GWAS

GWAS were performed for the six morphometric traits using PLINK [27]. A linear regression using an additive genetic model was applied, and defined as follows:

3 $$ \mathbf{y}=\mathbf{Xb}+\mathbf{Wg}+\mathbf{e} $$

where **y** was a vector of morphometric traits; **b** was a vector of fixed effects including Age, Sex, AEZ_Breed, and linear discriminant functions; **g** was a vector for the SNP effects; **e** was a vector of random residual effects with $\mathbf{e}\sim N\left(0,\mathbf{I}{\sigma}_e^2\right)$; and **X**, **W** were incidence matrices for **b** and **g**, respectively.

For a verification of results from PLINK, we additionally performed GWAS applying the following model (eq. 4) in the GCTA software [28]. However, considering the complexity of the mixed linear model in GCTA [28], and the small size of our dataset, only SNP detected by PLINK are prioritized and described. In matrix notation, the mixed model is:

4 $$ \mathbf{y}=\mathbf{Xb}+\mathbf{Wg}+\mathbf{Zu}+\mathbf{e} $$

where **y**, **g**, **e** and incidence matrices **X** and **W** were notations as defined in Eq. 3; **b** was a vector of fixed effects including Age, Sex, AEZ_Breed; **Z** and **u** were notations as defined in Eq. 1.

The assessment of the models for population stratification based on the genomic inflation factor (lambda-λ) and on the quantile–quantile (Q–Q) plot. Manhattan and Q-Q-plots plots were generated by means of the ggplot2 package in R [148].

Significantly associated SNP were detected according to the Bonferroni corrected significance threshold (*p* = 1.55 × 10^− 06^), i.e., calculated as *p = 0.05 / m,* with *m* = 32,185 (the effective number of SNP). In addition, chromosome-wide Bonferroni-corrected significance thresholds (*p*_*c*_ *= 0.05 / m*_*c*_*,*) with *m*_*c*_ denoting the effective number of SNP for each chromosome (see Additional file 5, Table S4) were used to identify suggestively associated variants. The effective numbers of SNP for the whole genome (*m*) and for each chromosome (*m*_*c*_) were determined from the genetic type I error calculator (GEC) [149].

### Candidate genes and functional annotations

To identify potential candidate genes associated with the morphometric traits, the rs-accession numbers of the significant and suggestive SNP were retrieved from the Ensembl genome database (version 96), using the BioMart R package [150, 151]. Genes were mapped to identified SNP, and only those located within a window frame of ±25 kb around each SNP were considered. In cases with more than two identified genes within the defined window frame, preference was given to the gene in which the SNP was located, or to the gene in closest SNP distance. However, if the SNP were located between two genes, both genes were selected, and the remaining genes were discarded. The gene mapping method as well as the window frame of ±25 kb were chosen to increase precision in selection of candidate genes.

Further, functional annotation was performed on the set of identified candidate genes per trait using the DAVID (see Additional file 6, Table S5 for the extensive outputs) [53]. In addition, pathways (KEGG and reactome pathways) and the biological process GO terms for candidate genes were retrieved manually, in order to infer potential gene functions.

## Supplementary Information

**Additional file 1: Figure S1.** Scatterplots for the first four linear discriminant functions (LDF) included as covariates in GWAS model for population stratification in the four Beninese indigenous cattle breeds. The coloring represents the original breed assignment of samples.

**Additional file 2: Table S1.** Characteristics and origins of sampled animals in four Beninese indigenous cattle breeds. Animal ID, sex, breed, year of sampling and origin: farm longitude and latitude, commune, agro-ecological zones (AEZ).

**Additional file 3: Figure S2.** The variations of six morphometric traits from all (ALL) and from four respective Beninese indigenous cattle. Height at withers (HAW), sacrum height (SH), heart girth (HG), hip width (HW), body length (BL) and ear length (EL).

**Additional file 4: Table S2.** Effects of AEZ_B, sex and age in multi-factor linear models on six morphometric traits in four Beninese indigenous cattle breeds. Analysis of variance (ANOVA) table presenting the significance of fixed effects on height at withers (HAW), sacrum height (SH), heart girth (HG), hip width (HW), body length (BL) and ear length (EL). **Table S3.** Genome-wide and chromosome-wide significant SNP associated with six morphometric traits from GWAS model including the first four principal components (instead of the linear discriminant functions as considered for main results).

**Additional file 5: Table S4.** Chromosome-wide significance thresholds used in multi-breed GWAS for conformation traits in four Beninese indigenous cattle breeds.

**Additional file 6: Table S5.** Functional annotation of candidate genes for six morphometric traits in four Beninese indigenous cattle breeds. Results retrieved from the database for annotation, visualization and integrated discovery (DAVID) for candidate genes associated with height at withers (HAW), sacrum height (SH), heart girth (HG), hip width (HW), body length (BL) and ear length (EL).

## Data Availability

All the data supporting the results of this article are presented within the article or in the additional files. The raw phenotypic and genotypic data are stored in the cloud of the University of Giessen (https://jlubox.uni-giessen.de) and can be provided by the corresponding author on reasonable request.

## Abbreviations

AEZ: Agro-ecological zone
ANOVA: Analysis of variance
BL: Body length
DAPC: Discriminant analysis of principal components
DAVID: Database for annotation, visualization and integrated discovery
EL: Ear length
FAO: Food and agriculture organization of the United Nations
*GCTA*: Genome-wide complex trait analysis
GEC: Genetic Type I error calculator
GWAS: Genome-wide association study
HAW: Height at withers
HG: Heart girth
HW: Hip width
kb: Kilo base pairs = 1000 base pairs
KEGG: Kyoto Encyclopedia of Genes and Genomes
LD: Linkage disequilibrium
LDF: Linear discriminant functions
Mb: Mega bases pairs = 1000 kb = 1 million base pairs
PCA: Principal component analysis
REML: Restricted maximum likelihood
r_g_: Genetic correlations
r_p_: Phenotypic correlation
SH: Sacrum height
SNP: Single nucleotide polymorphism
