# Supplementary material for: A multi-breed GWAS for morphometric traits in four Beninese indigenous cattle breeds reveals loci associated with conformation, carcass and adaptive traits
Source: BMC Genomics. 2020 Nov 11;21:783. doi: 10.1186/s12864-020-07170-0 (PMC7656759; doi:10.1186/s12864-020-07170-0)
Supplement: Supplementary file 1 — Additional file 1: Figure S1. Scatterplots for the first four linear discriminant functions (LDF) included as covariates in GWAS model for population stratification in the four Beninese indigenous cattle breeds. The coloring represents the original breed assignment of samples. [file 12864_2020_7170_MOESM1_ESM.pdf]

Additional file 1

Figure S1. Scatterplots for the first four linear discriminant functions describing the population structure of the four Beninese indigenous cattle breeds.

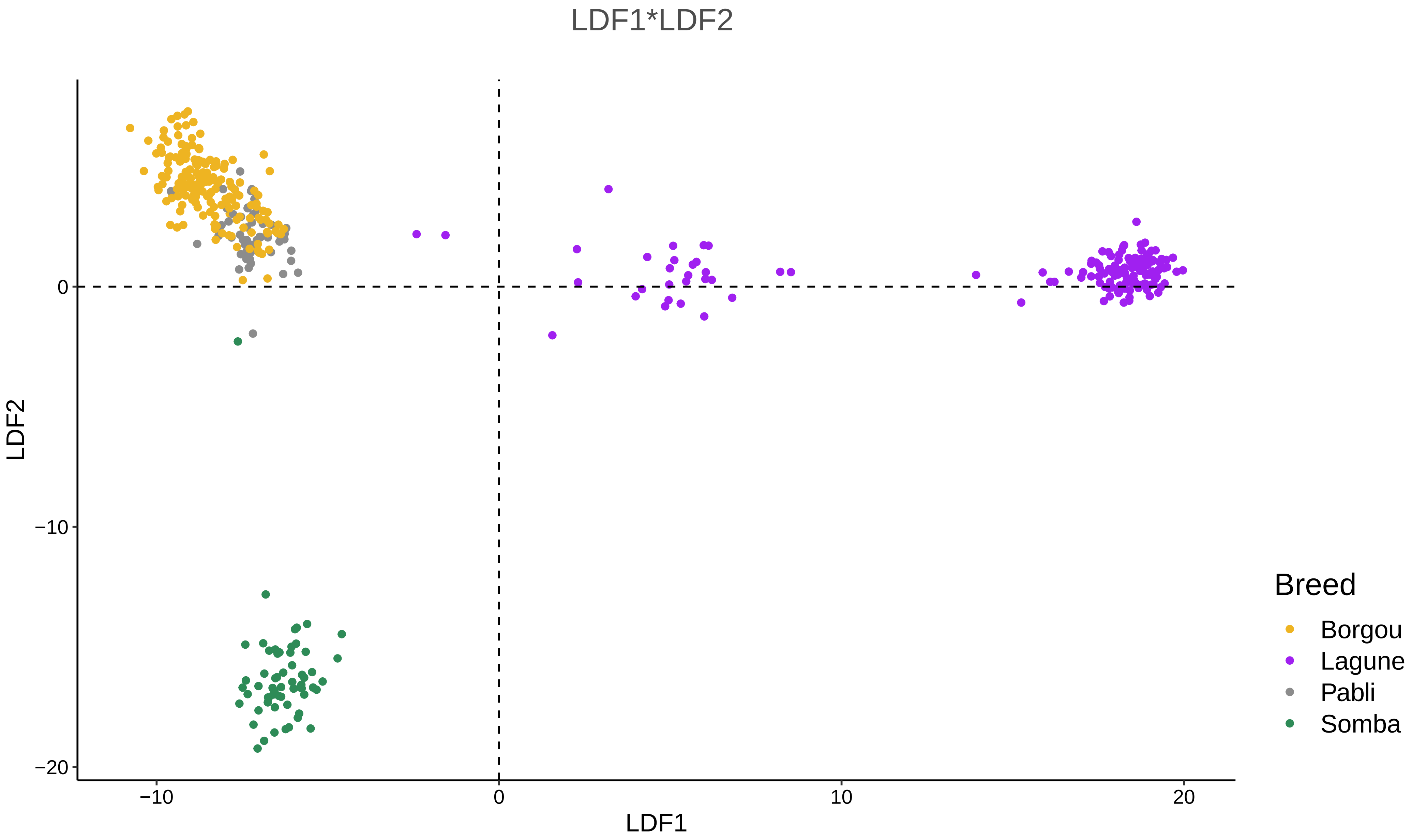

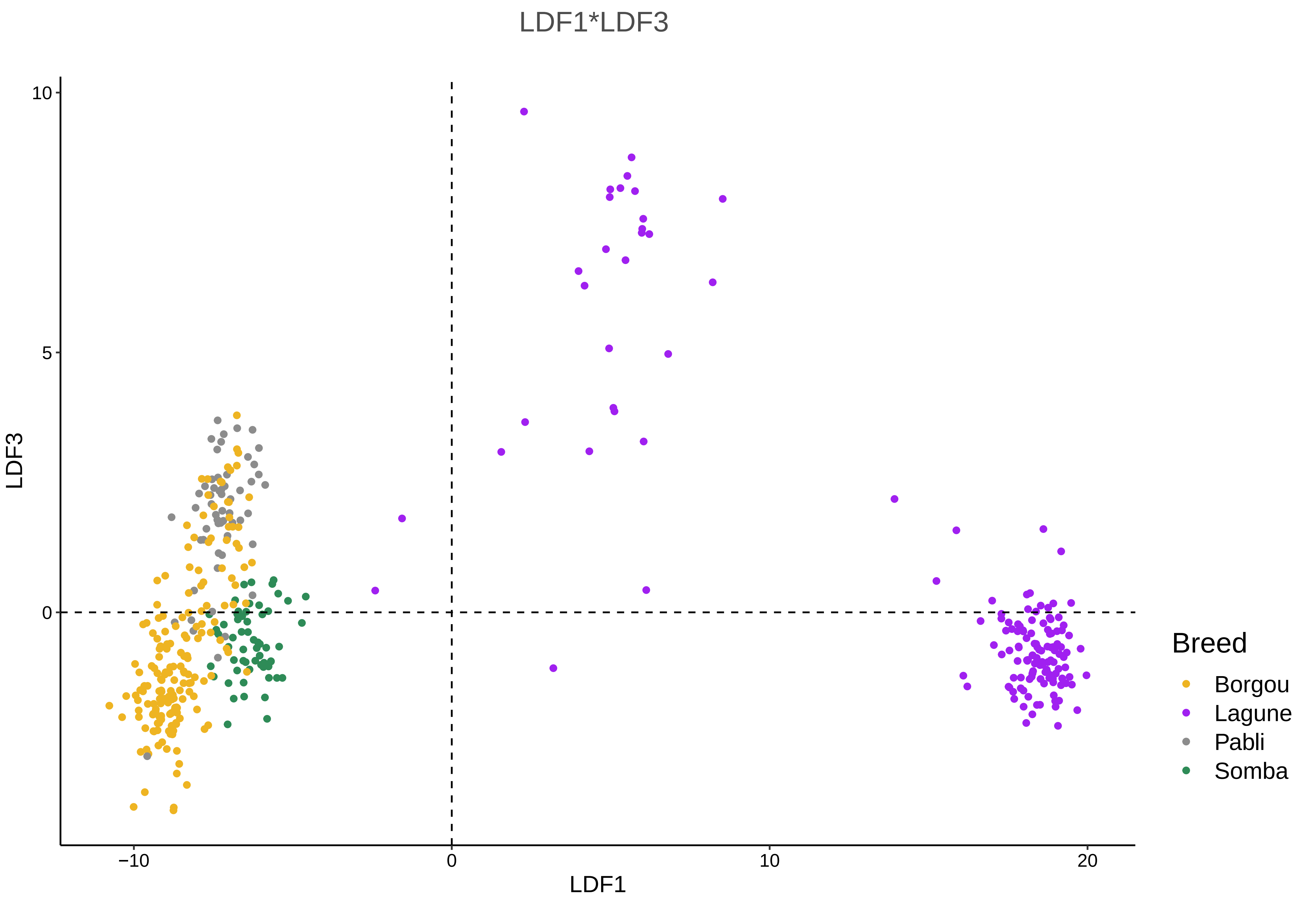
LDF1*LDF3
LDF3
LDF1
10
5
0
−10
0
10
20
Breed
Borgou
Lagune
Pabli
Somba

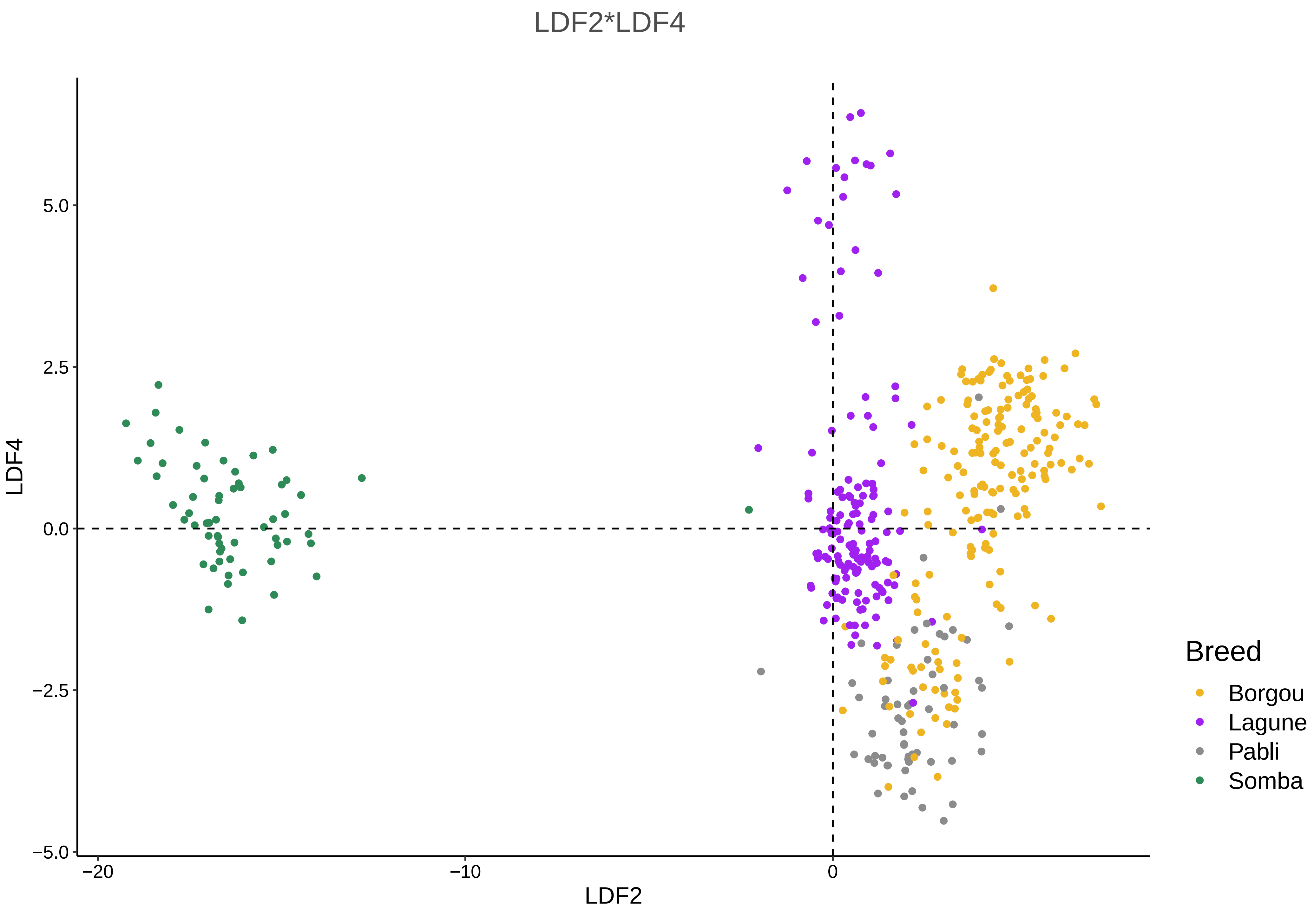

LDF2*LDF4
LDF4
5.0
2.5
0.0
−2.5
−5.0
−20
−10
0
LDF2
Breed
Borgou
Lagune
Pabli
Somba
