# Supplementary material for: A multi-breed GWAS for morphometric traits in four Beninese indigenous cattle breeds reveals loci associated with conformation, carcass and adaptive traits
Source: BMC Genomics. 2020 Nov 11;21:783. doi: 10.1186/s12864-020-07170-0 (PMC7656759; doi:10.1186/s12864-020-07170-0)
Supplement: Supplementary file 3 — Additional file 3: Figure S2. The variations of six morphometric traits from all (ALL) and from four respective Beninese indigenous cattle. Height at withers (HAW), sacrum height (SH), heart girth (HG), hip width (HW), body length (BL) and ear length (EL). [file 12864_2020_7170_MOESM3_ESM.pdf]

Additional file 3
Figure S2. Boxplots presenting the variation of six morphometric traits from all and from four Beninese indigenous cattle

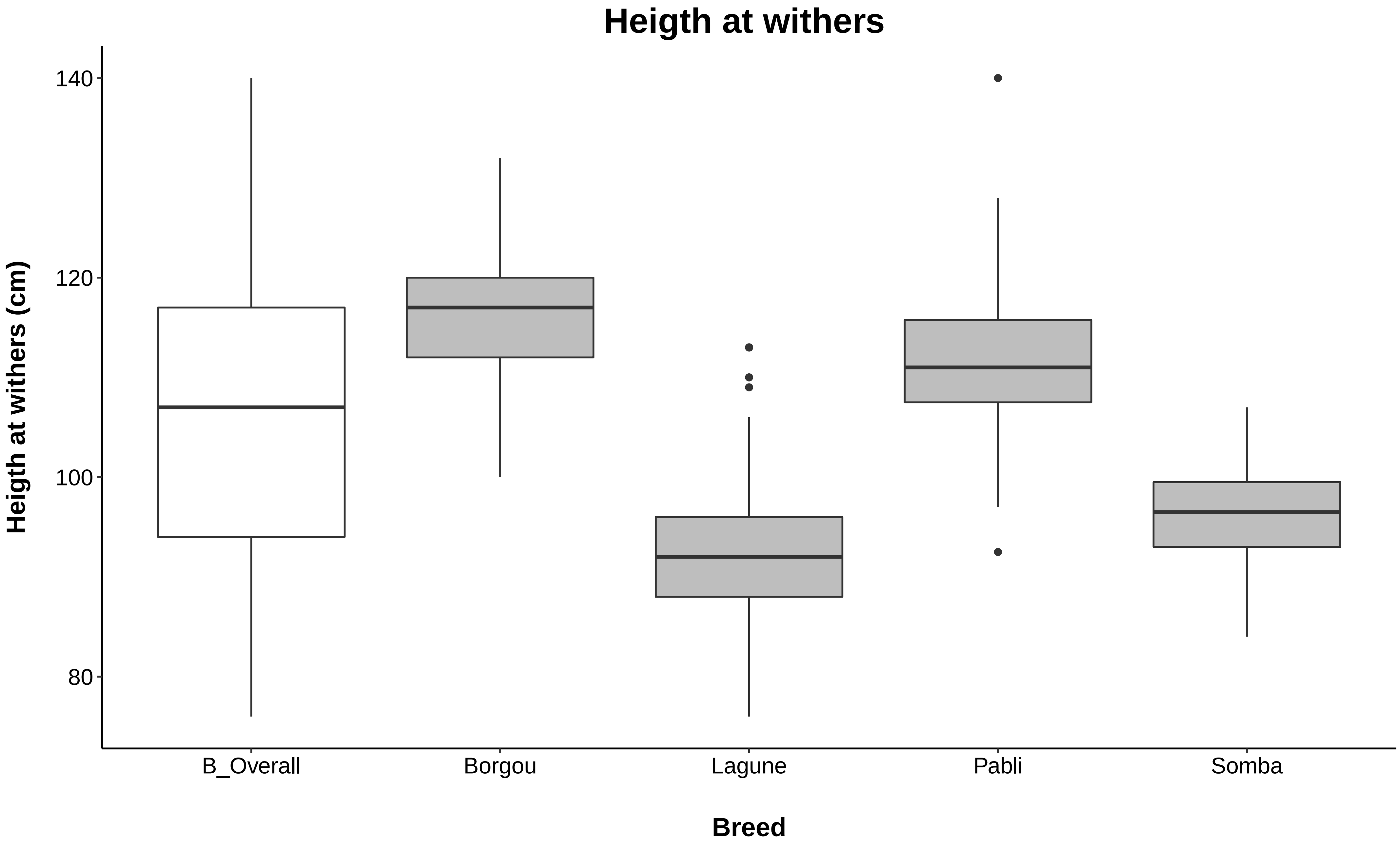

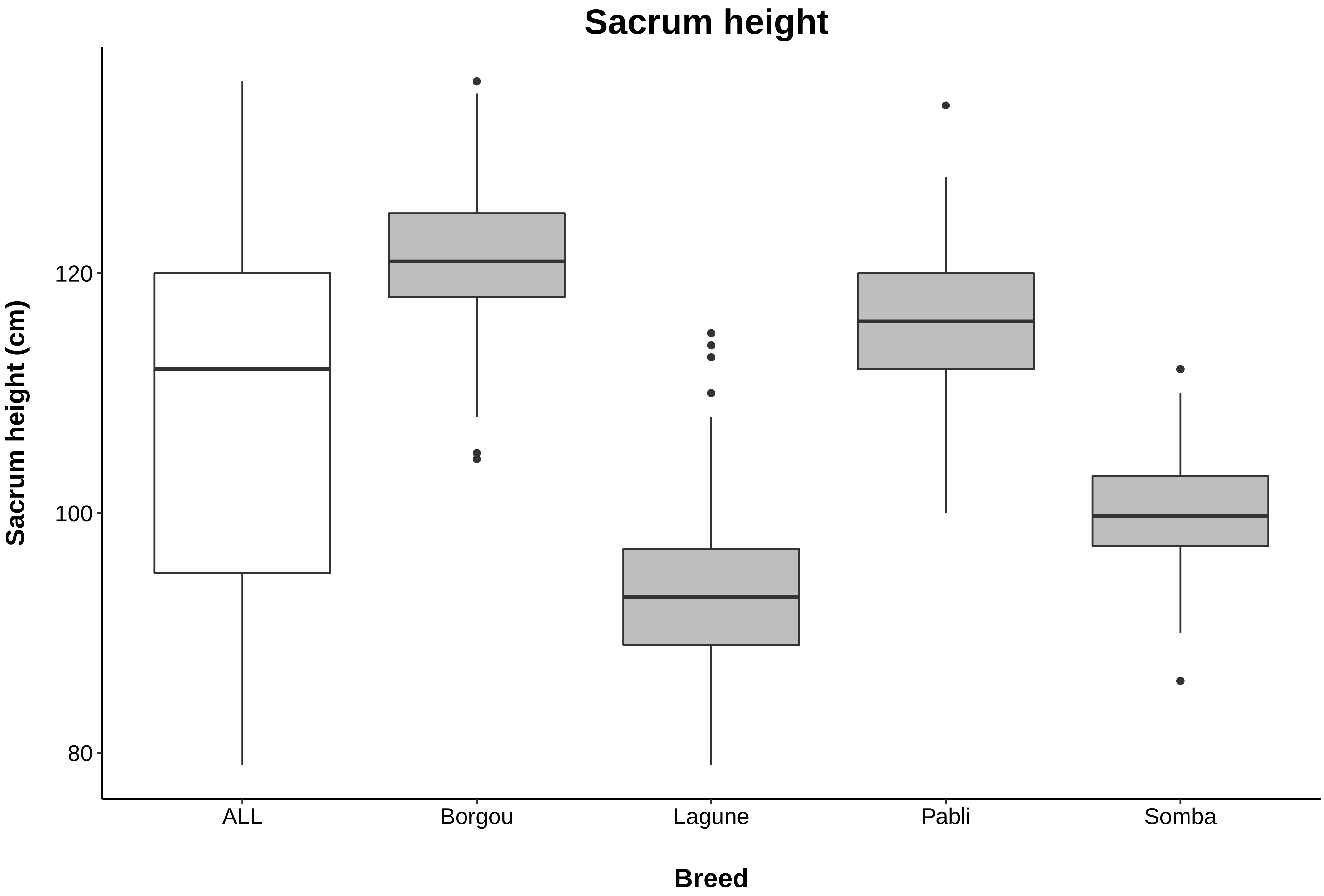
Sacrum height
Sacrum height (cm)
120
100
80
ALL
Borgou
Lagune
Pabli
Somba
Breed

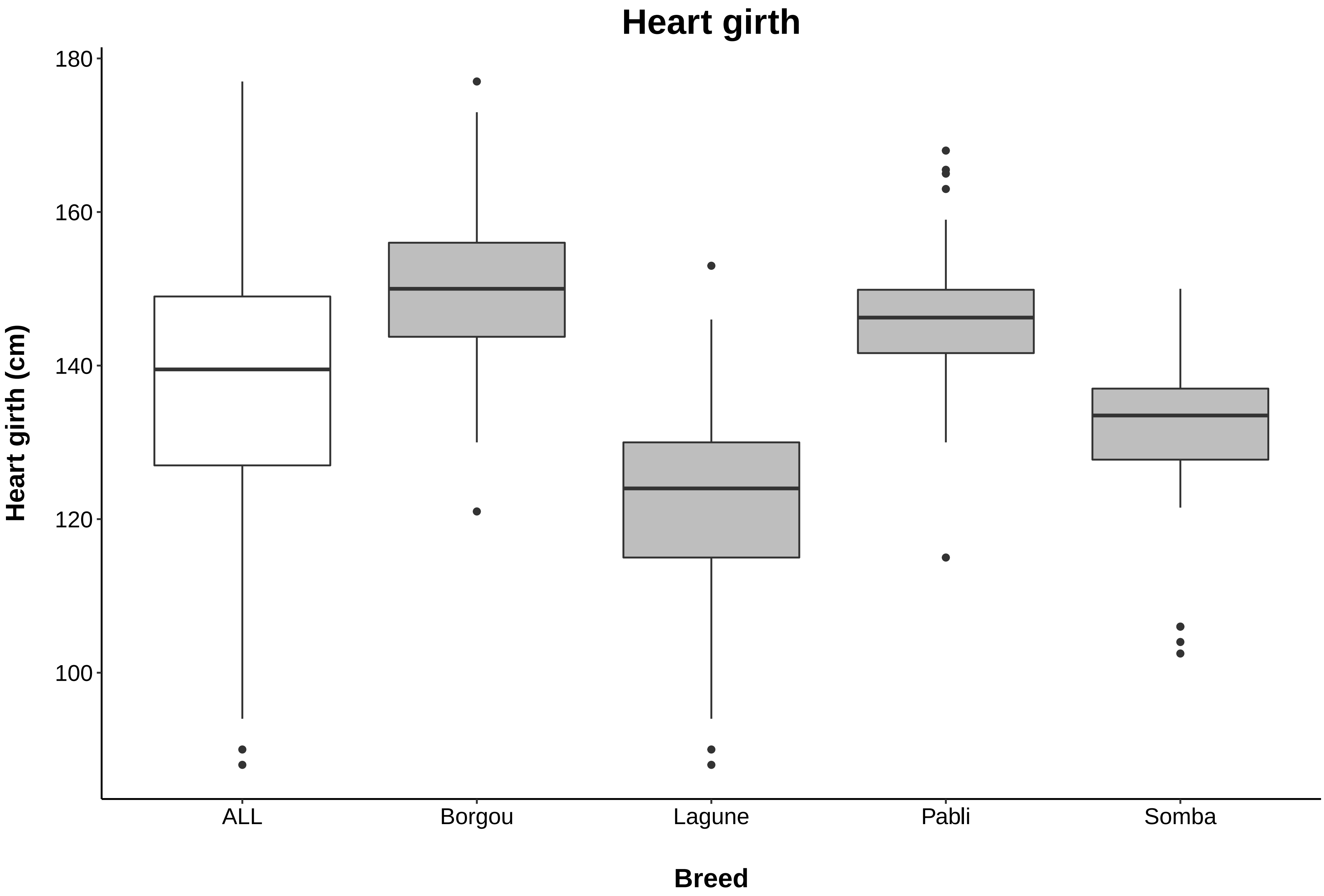

Heart girth
180
160
140
120
100
Heart girth (cm)
ALL
Borgou
Lagune
Pabli
Somba
Breed

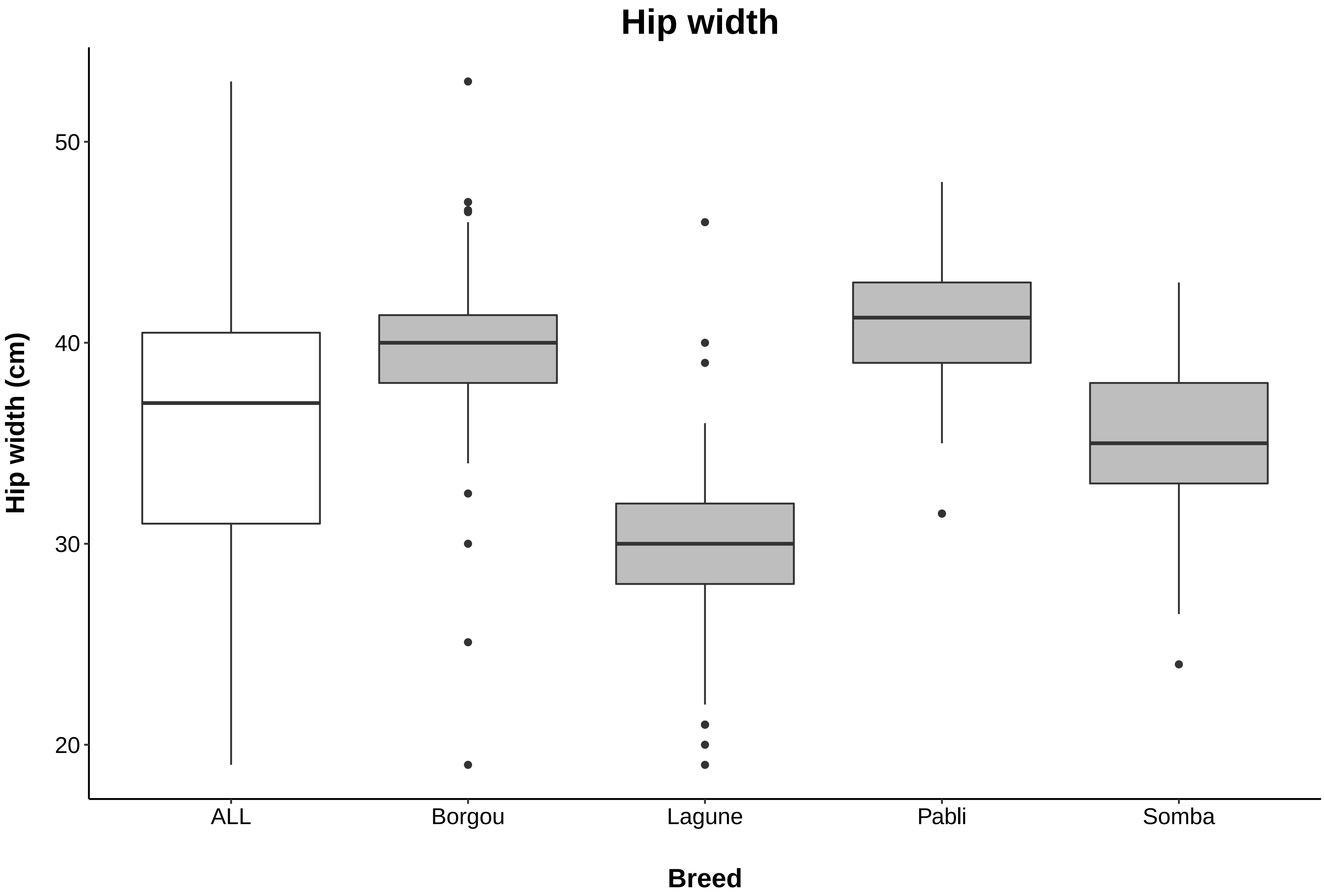
Hip width
Hip width (cm)
50
40
30
20
ALL
Borgou
Lagune
Pabli
Somba
Breed

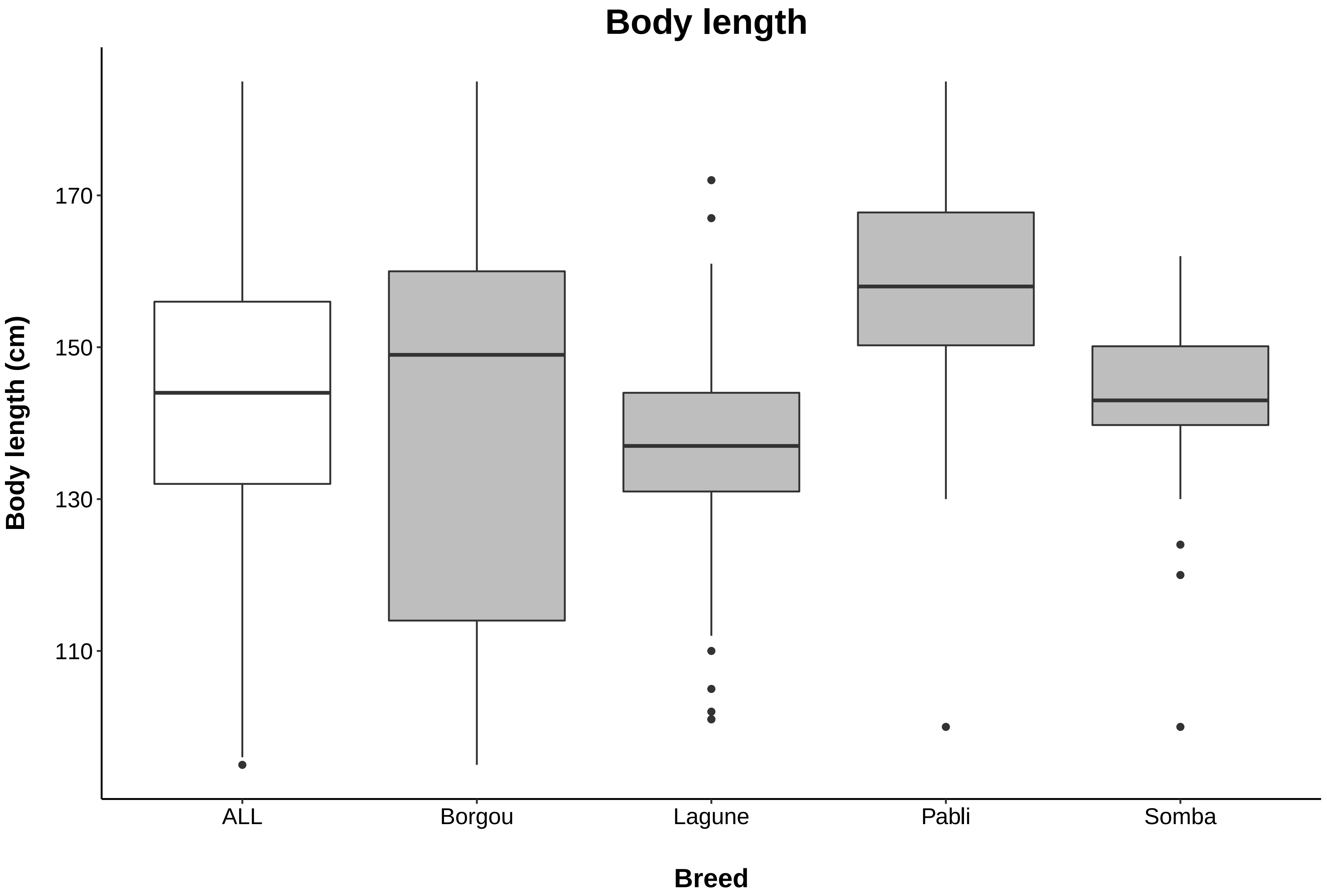

Body length
Body length (cm)
170
150
130
110
ALL
Borgou
Lagune
Pabli
Somba
Breed

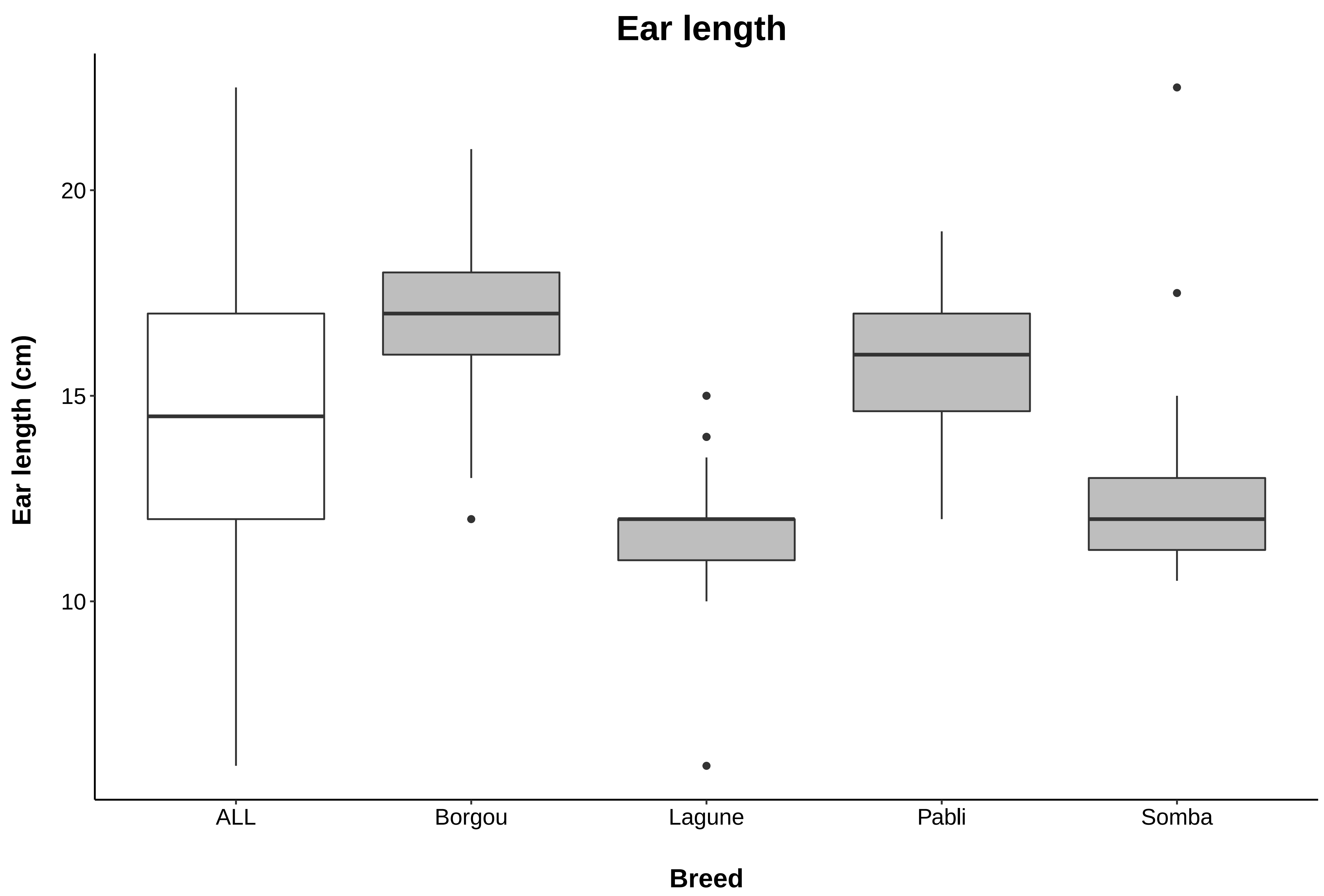

Ear length
Ear length (cm)
20
15
10
ALL
Borgou
Lagune
Pabli
Somba
Breed
