# Supplementary material for: A multi-breed GWAS for morphometric traits in four Beninese indigenous cattle breeds reveals loci associated with conformation, carcass and adaptive traits
Source: BMC Genomics. 2020 Nov 11;21:783. doi: 10.1186/s12864-020-07170-0 (PMC7656759; doi:10.1186/s12864-020-07170-0)
Supplement: Supplementary file 4 — Additional file 4: Table S2. Effects of AEZ_B, sex and age in multi-factor linear models on six morphometric traits in four Beninese indigenous cattle breeds. Analysis of variance (ANOVA) table presenting the significance of fixed effects on height at withers (HAW), sacrum height (SH), heart girth (HG), hip width (HW), body length (BL) and ear length (EL). Table S3. Genome-wide and chromosome-wide significant SNP associated with six morphometric traits from GWAS model including the first four principal components (instead of the linear discriminant functions as considered for main results). [file 12864_2020_7170_MOESM4_ESM.pdf]

Additional file 4

Table S2 Effects of AEZ_B, sex and age in multi-factor linear model on six morphometric traits in four Beninese indigenous cattle.

	Sum Sq	Df	F value	Pr(>F)
**Height at withers (HAW)**				
Intercept	265975.270	1.000	6285.250	<0.001
Factor (AEZ_B)	53962.940	7.000	182.170	<0.001
Factor (Sex)	26.460	1.000	0.630	0.430
Age	259.990	1.000	6.140	0.014
Residuals	18069.520	427.000		
**Sacrum height**				
Intercept	287259.410	1.000	3141.540	<0.001
Factor (AEZ_B)	60479.940	7.000	94.490	<0.001
Factor (Sex)	92.850	1.000	1.020	0.314
Age	501.760	1.000	5.490	0.020
Residuals	39044.440	427.000		
**Heart girth (HG)**				
Intercept	378575.000	1.000	1528.550	<0.001
Factor (AEZ_B)	58998.850	7.000	34.030	<0.001
Factor (Sex)	1236.140	1.000	4.990	0.026
Age	3583.910	1.000	14.470	<0.001
Residuals	106002.290	428.000		
**Hip width (HW)**				
Intercept	30180.600	1.000	1203.300	<0.001
Factor (AEZ_B)	8647.390	7.000	49.250	<0.001
Factor (Sex)	334.850	1.000	13.350	<0.001
Age	126.650	1.000	5.050	0.025
Residuals	10734.880	428.000		
**Body length (BL)**				
Intercept	502708.810	1.000	1457.270	<0.001
Factor (AEZ_B)	46873.390	7.000	19.410	<0.001
Factor (Sex)	2994.750	1.000	8.680	0.003
Age	18.520	1.000	0.050	0.817
Residuals	147645.580	428.000		

	Sum Sq	Df	F value	Pr(>F)
**Ear length (EL)**				
Intercept	8710.580	1.000	101.670	<0.001
Factor (AEZ_B)	3013.990	7.000	5.030	<0.001
Factor (Sex)	147.020	1.000	1.720	0.191
Age	146.680	1.000	1.710	0.191
Residuals	36496.380	426.000		

Table S3. Genome-wide and chromosome-wide significant SNP associated with six morphometric traits from GWAS model including the first four principal components (instead of linear discriminat functions)

	CHR	SNP	BP	A1	BETA	STAT	P	sig_chr
**Height at withers**								
	1	ARS-BFGL-NGS-57889	110160486	A	2,148	4,805	2,149E-06	2,4399E-05
	3	ARS-BFGL-NGS-31952	81139242	C	2,538	4,555	6,833E-06	3,2869E-05
	16	BTA-23170-no-rs	29174251	C	1,974	4,242	0,00002715	4,7255E-05
	17	Hapmap50686-BTA-41836	68037062	G	2,543	4,201	0,00003238	4,9302E-05
**Sacrum height**								
	1	BTB-01585499	157367219	A	1,948	4,375	0,00001527	2,4399E-05
	7	ARS-BFGL-NGS-7310	42545291	A	1,553	4,49	9,185E-06	3,1684E-05
	14	ARS-BFGL-BAC-1180	20574088	C	-1,58	-4,209	0,0000313	4,6687E-05
**Heart girth**								
	2	ARS-BFGL-NGS-77689	32163209	C	2,9	4,543	7,234E-06	3,023E-05
	19	Hapmap48676-BTA-18047	46729603	A	2,58	4,117	0,00004614	5,7291E-05
	22	Hapmap39844-BTA-54797	48208654	C	-3,443	-4,111	0,0000472	6,3571E-05
**Hip width**								
	6	BTA-07718-rs29027383	38646790	G	-4,159	-4,366	1,59E-05	2,83E-05
	8	ARS-BFGL-NGS-119529	103907956	T	-3,141	-4,352	1,69E-05	3,49E-05
	16	BTB-01732320	60525984	C	-1,144	-4,417	1,27E-05	4,73E-05
**Body length**								
	2	ARS-BFGL-NGS-109828	76610609	C	-16,19	-4,471	9,989E-06	3,023E-05
	2	BTB-01145402	113155761	G	-5,635	-4,547	7,105E-06	3,023E-05
	3	BovineHD0300010335	33048892	G	-7,095	-4,375	0,00001526	3,2869E-05

	CHR	SNP	BP	A1	BETA	STAT	P	sig_chr
	3	BovineHD0300010338	33060757	A	-6,803	-4,219	0,00002993	3,2869E-05
	21	Hapmap33092-BTA-51753	18775375	T	-10,38	-5,187	3,301E-07	5,5988E-05
	23	Hapmap57845-rs29014813	33230735	C	-6,283	-4,27	0,00002407	6,616E-05
	27	Hapmap44720-BTA-62525	24546315	G	-6,068	-4,139	0,00004203	7,9641E-05
**Ear length**								
	20	ARS-BFGL-NGS-44763	40060386	T	0,5675	4,369	0,00001569	5,1176E-05
