# Supplementary material for: A multi-breed GWAS for morphometric traits in four Beninese indigenous cattle breeds reveals loci associated with conformation, carcass and adaptive traits
Source: BMC Genomics. 2020 Nov 11;21:783. doi: 10.1186/s12864-020-07170-0 (PMC7656759; doi:10.1186/s12864-020-07170-0)
Supplement: Supplementary file 5 — Additional file 5: Table S4. Chromosome-wide significance thresholds used in multi-breed GWAS for conformation traits in four Beninese indigenous cattle breeds. [file 12864_2020_7170_MOESM5_ESM.pdf]

**Additional file 5**

**Table S4: Chromosome-wide significance thresholds used in multibreed GWAS for conformation traits in four Beninese indigenous cattle**

| **BTA** | **SNP marker after QC** | **Effective number of independent SNP marker based on LD calculated using GEC** | **p-value threshold ($p_C$)** |
|---|---|---|---|
| 1 | 2376 | 2049.3 | 2.44E-05 |
| 2 | 1893 | 1654 | 3.02E-05 |
| 3 | 1738 | 1521.19 | 3.29E-05 |
| 4 | 1710 | 1520.59 | 3.29E-05 |
| 5 | 1453 | 1281.61 | 3.90E-05 |
| 6 | 2156 | 1764.76 | 2.83E-05 |
| 7 | 1855 | 1578.1 | 3.17E-05 |
| 8 | 1635 | 1433.32 | 3.49E-05 |
| 9 | 1493 | 1310.28 | 3.82E-05 |
| 10 | 1654 | 1411.28 | 3.54E-05 |
| 11 | 1518 | 1333.92 | 3.75E-05 |
| 12 | 1163 | 1043.83 | 4.79E-05 |
| 13 | 1184 | 1063.92 | 4.70E-05 |
| 14 | 1200 | 1070.96 | 4.67E-05 |
| 15 | 1177 | 1049.68 | 4.76E-05 |
| 16 | 1199 | 1058.1 | 4.73E-05 |
| 17 | 1157 | 1014.16 | 4.93E-05 |
| 18 | 914 | 824.35 | 6.07E-05 |
| 19 | 984 | 872.74 | 5.73E-05 |
| 20 | 1154 | 977.02 | 5.12E-05 |
| 21 | 1022 | 893.05 | 5.60E-05 |
| 22 | 874 | 786.52 | 6.36E-05 |
| 23 | 855 | 755.74 | 6.62E-05 |
| 24 | 878 | 776.26 | 6.44E-05 |
| 25 | 647 | 585.93 | 8.53E-05 |
| 26 | 718 | 648.8 | 7.71E-05 |
| 27 | 693 | 627.82 | 7.96E-05 |
| 28 | 674 | 617.25 | 8.10E-05 |
| 29 | 746 | 660.34 | 7.57E-05 |
| **overall** | **36720** | **32184.82** | **1.55E-06** |
