# Supplementary material for: A multi-breed GWAS for morphometric traits in four Beninese indigenous cattle breeds reveals loci associated with conformation, carcass and adaptive traits
Source: BMC Genomics. 2020 Nov 11;21:783. doi: 10.1186/s12864-020-07170-0 (PMC7656759; doi:10.1186/s12864-020-07170-0)
Supplement: Supplementary file 6 — Additional file 6: Table S5. Functional annotation of candidate genes for six morphometric traits in four Beninese indigenous cattle breeds. Results retrieved from the database for annotation, visualization and integrated discovery (DAVID) for candidate genes associated with height at withers (HAW), sacrum height (SH), heart girth (HG), hip width (HW), body length (BL) and ear length (EL). [file 12864_2020_7170_MOESM6_ESM.pdf]

Additional file 6

Table S5. Functional annotation of candidate genes for six morphometric traits in four Beninese indigenous cattle. Results retrieved from the database for annotation, visualization and integrated discovery (DAVID) for genes associated with height at withers (HAW), sacrum height (SH), heart girth (HG), hip width (HW), body length (BL) and ear length (EL).

**Height at withers (HAW)**	
ENSBTAG00000003557	***coiled-coil domain containing 117(CCDC117)***
CHROMOSOME	17, Un,
ENSEMBL_GENE_ID	ENSBTAG00000003557,
PUBMED_ID	19393038,
UP_KEYWORDS	Complete proteome, Reference proteome,
UP_TISSUE	Ascending colon,
ENSBTAG00000016344	***phosphoinositide-3-kinase regulatory subunit 6(PIK3R6)***
CHROMOSOME	19,
ENSEMBL_GENE_ID	ENSBTAG00000016344,
GOTERM_BP_DIRECT	G-protein coupled receptor signaling pathway, phosphatidylinositol 3-kinase signaling, regulation of natural killer cell mediated cytotoxicity, positive regulation of MAP kinase activity, positive regulation of T cell differentiation, positive regulation of angiogenesis,
GOTERM_CC_DIRECT	intracellular, phosphatidylinositol 3-kinase complex, phosphatidylinositol 3-kinase complex, class IB,
GOTERM_MF_DIRECT	phosphatidylinositol-4,5-bisphosphate 3-kinase activity, 1-phosphatidylinositol-3-kinase regulator activity,
INTERPRO	Phosphoinositide 3-kinase 1B, gamma adapter, p101 subunit,
PFAM	Phosphoinositide 3-kinase gamma adapter protein p101 subunit,
PUBMED_ID	19393038,
REACTOME_PATHWAY	R-BTA-114604, R-BTA-1660499, R-BTA-392451,
UP_KEYWORDS	Complete proteome, Reference proteome,
UP_TISSUE	Kidney,
ENSBTAG00000011011	***slingshot protein phosphatase 2(SSH2)***
CHROMOSOME	19,
ENSEMBL_GENE_ID	ENSBTAG00000011011,
GOTERM_BP_DIRECT	regulation of actin polymerization or depolymerization, regulation of lamellipodium assembly, regulation of axonogenesis,
GOTERM_CC_DIRECT	cytoplasm, cytoskeleton,
GOTERM_MF_DIRECT	DNA binding, actin binding, protein tyrosine phosphatase activity, protein tyrosine/serine/threonine phosphatase activity,
INTERPRO	Dual specificity phosphatase, catalytic domain, Protein-tyrosine/Dual specificity phosphatase, Homeodomain-like, DEK, C-terminal, Protein-tyrosine phosphatase, active site, Dual specificity phosphatase, subgroup, catalytic domain, Dual specificity phosphatase, Protein phosphatase Slingshot,
KEGG_PATHWAY	Regulation of actin cytoskeleton,

PFAM	Dual specificity phosphatase, catalytic domain, DEK C terminal domain,
PUBMED_ID	19393038,
SMART	DSPc,
UP_KEYWORDS	Complete proteome, Reference proteome,
ENSBTAG00000024643	***ventricular zone expressed PH domain containing 1(VEPH1)***
ENSEMBL_GENE_ID	ENSBTAG00000024643,
ENTREZ_GENE_ID	100337421,
INTERPRO	Pleckstrin homology domain, Pleckstrin homology-like domain, Armadillo-type fold,
PFAM	PH domain,
PUBMED_ID	19393038,
SMART	PH,
UP_KEYWORDS	Complete proteome, Reference proteome,
**Sacrum height (SH)**	
ENSBTAG00000030711	***LY6/PLAUR domain containing 8(LYPD8)***
CHROMOSOME	7,
ENSEMBL_GENE_ID	ENSBTAG00000030711,
GOTERM_BP_DIRECT	defense response to Gram-negative bacterium,
GOTERM_CC_DIRECT	extracellular space, plasma membrane, anchored component of membrane,
INTERPRO	Ly-6 antigen / uPA receptor -like,
PFAM	u-PAR/Ly-6 domain,
UP_KEYWORDS	Cell membrane, Complete proteome, Glycoprotein, GPI-anchor, Lipoprotein, Membrane, Reference proteome, Secreted, Signal,
UP_SEQ_FEATURE	chain:Uncharacterized protein UNQ511/PRO1026 homolog, glycosylation site:N-linked (GlcNAc...), lipid moiety-binding region:GPI-anchor amidated serine, propeptide:Removed in mature form, signal peptide,
UP_TISSUE	Ileum,
ENSBTAG00000010989	***phosphoinositide-3-kinase regulatory subunit 1(PIK3R1)***
BIOGRID_INTERACTION	442985:PDGFRB~platelet derived growth factor receptor beta,
CHROMOSOME	20,
COG_ONTOLOGY	Cell division and chromosome partitioning,
ENSEMBL_GENE_ID	ENSBTAG00000010989,
GENERIF_SUMMARY	The effect of PIA [N6-(2-phenylisopropyl)adenosine] in p110alpha-deficient cells was attenuated effectively by both Deltap85 and betaARK-CT (beta-adrenergic receptor kinase-C-terminal peptide)., The domains of p85alpha responsible for the interaction with the FTK protein network and transduction of leukemogenic signaling were identified., The p85alpha regulatory subunit of PI3K mediates cAMP-PKA and retinoic acid biological effects on MCF7 cell growth and migration.,
GOTERM_BP_DIRECT	cellular glucose homeostasis, negative regulation of cell-matrix adhesion, protein phosphorylation, insulin receptor signaling pathway, extrinsic apoptotic signaling pathway via death domain receptors, intrinsic apoptotic signaling pathway in response to DNA damage, phosphatidylinositol 3-kinase signaling, B cell differentiation, positive regulation of cell migration, positive regulation of tumor necrosis factor production, cellular response to insulin stimulus, positive regulation of RNA splicing, cellular response to UV, response to endoplasmic reticulum stress,

	positive regulation of transcription factor import into nucleus, negative regulation of apoptotic process, regulation of phosphatidylinositol 3-kinase activity, negative regulation of osteoclast differentiation, positive regulation of transcription from RNA polymerase II promoter, positive regulation of glucose import, phosphatidylinositol phosphorylation, insulin-like growth factor receptor signaling pathway, protein stabilization, regulation of stress fiber assembly, NFAT protein import into nucleus, growth hormone receptor signaling pathway, positive regulation of establishment of protein localization to plasma membrane, positive regulation of endoplasmic reticulum unfolded protein response, positive regulation of glucose import in response to insulin stimulus,
GOTERM_CC_DIRECT	nucleus, cis-Golgi network, cytosol, cell-cell junction, phosphatidylinositol 3-kinase complex, phosphatidylinositol 3-kinase complex, class IA, perinuclear endoplasmic reticulum membrane,
GOTERM_MF_DIRECT	transmembrane receptor protein tyrosine kinase adaptor activity, insulin receptor binding, insulin-like growth factor receptor binding, neurotrophin TRKA receptor binding, protein binding, transcription factor binding, protein phosphatase binding, phosphatidylinositol 3-kinase regulator activity, phosphatidylinositol 3-kinase regulatory subunit binding, ErbB-3 class receptor binding, phosphatidylinositol 3-kinase binding, insulin binding, insulin receptor substrate binding, 1-phosphatidylinositol-3-kinase regulator activity, protein heterodimerization activity,
INTACT	282306:phosphatidylinositol-4,5-bisphosphate 3-kinase catalytic subunit alpha(PIK3CA),
INTERPRO	Rho GTPase-activating protein domain, SH2 domain, Src homology-3 domain, PI3 kinase, P85 regulatory subunit, Rho GTPase activation protein,
KEGG_PATHWAY	ErbB signaling pathway, Ras signaling pathway, Rap1 signaling pathway, cAMP signaling pathway, Chemokine signaling pathway, HIF-1 signaling pathway, FoxO signaling pathway, Phosphatidylinositol signaling system, Sphingolipid signaling pathway, mTOR signaling pathway, PI3K-Akt signaling pathway, AMPK signaling pathway, Apoptosis, VEGF signaling pathway, Osteoclast differentiation, Focal adhesion, Signaling pathways regulating pluripotency of stem cells, Platelet activation, Toll-like receptor signaling pathway, Jak-STAT signaling pathway, Natural killer cell mediated cytotoxicity, T cell receptor signaling pathway, B cell receptor signaling pathway, Fc epsilon RI signaling pathway, Fc gamma R-mediated phagocytosis, TNF signaling pathway, Leukocyte transendothelial migration, Neurotrophin signaling pathway, Cholinergic synapse, Inflammatory mediator regulation of TRP channels, Regulation of actin cytoskeleton, Insulin signaling pathway, Progesterone-mediated oocyte maturation, Estrogen signaling pathway, Prolactin signaling pathway, Thyroid hormone signaling pathway, Regulation of lipolysis in adipocytes, Type II diabetes mellitus, Insulin resistance, Non-alcoholic fatty liver disease (NAFLD), Aldosterone-regulated sodium reabsorption, Carbohydrate digestion and absorption, Bacterial invasion of epithelial cells, Chagas disease (American trypanosomiasis), Amoebiasis,

	Hepatitis C, Hepatitis B, Measles, Influenza A, HTLV-I infection, Epstein-Barr virus infection, Pathways in cancer, Viral carcinogenesis, Proteoglycans in cancer, Colorectal cancer, Renal cell carcinoma, Pancreatic cancer, Endometrial cancer, Glioma, Prostate cancer, Melanoma, Chronic myeloid leukemia, Acute myeloid leukemia, Small cell lung cancer, Non-small cell lung cancer, Central carbon metabolism in cancer, Choline metabolism in cancer,
PFAM	SH2 domain, RhoGAP domain,
PUBMED_ID	10068665, 10336465, 1322797, 1323062, 1330535, 1372092, 15377662, 15680399, 15734648, 16091017, 16135792, 1707345, 19393038, 22366926, 24731625, 7505116, 7537265, 7683666, 7684655, 8052599, 8313896, 8382774, 8952511, 9512716,
REACTOME_PATHWAY	R-BTA-109704, R-BTA-114604, R-BTA-1250342, R-BTA-1257604, R-BTA-1433557, R-BTA-1660499, R-BTA-180292, R-BTA-186763, R-BTA-1963642, R-BTA-198203, R-BTA-202424, R-BTA-2029485, R-BTA-210993, R-BTA-2424491, R-BTA-2730905, R-BTA-388841, R-BTA-389357, R-BTA-392451, R-BTA-416476, R-BTA-416482, R-BTA-430116, R-BTA-4420097, R-BTA-512988, R-BTA-5654689, R-BTA-5654695, R-BTA-5654710, R-BTA-5654720, R-BTA-912526, R-BTA-912631, R-BTA-983695,
SMART	SH2, RhoGAP, SH3,
UP_KEYWORDS	3D-structure, Acetylation, Complete proteome, Phosphoprotein, Protein transport, Reference proteome, Repeat, SH2 domain, SH3 domain, Stress response, Transport, Ubl conjugation,
UP_SEQ_FEATURE	chain:Phosphatidylinositol 3-kinase regulatory subunit alpha, domain:Rho-GAP, domain:SH2 1, domain:SH2 2, domain:SH3, helix, modified residue, strand, turn,
**Heart girth (HG)**	
ENSBTAG00000043989	***EYA transcriptional coactivator and phosphatase 3(EYA3)***
CHROMOSOME	2,
EC_NUMBER	3.1.3.48,
ENSEMBL_GENE_ID	ENSBTAG00000043989,
GOTERM_BP_DIRECT	double-strand break repair, regulation of transcription, DNA-templated, multicellular organism development, response to ionizing radiation, histone dephosphorylation, cell differentiation, positive regulation of DNA repair, anatomical structure development, negative regulation of extrinsic apoptotic signaling pathway in absence of ligand,
GOTERM_CC_DIRECT	nucleus, nucleoplasm, transcription factor complex, centrosome,
GOTERM_MF_DIRECT	protein tyrosine phosphatase activity, metal ion binding,
INTERPRO	EYA,
PUBMED_ID	19393038,
REACTOME_PATHWAY	R-BTA-5693565,
UP_KEYWORDS	Complete proteome, Hydrolase, Magnesium, Metal-binding, Protein phosphatase, Reference proteome, Transcription, Transcription regulation,
ENSBTAG00000027051	***platelet activating factor receptor(PTAFR)***
CHROMOSOME	2,
ENSEMBL_GENE_ID	ENSBTAG00000027051,

GENERIF_SUMMARY	The results provide clear evidence for expression of PAFr in bovine granulosa cells and its functional involvement in PAF/PAFr-mediated stimulation of cell recruitment., PAF and luteinizing hormone signaling plays an important role in regulating the production of excessive oxidants.,
GOTERM_BP_DIRECT	cytokine production, chemotaxis, inflammatory response, female pregnancy, lipopolysaccharide-mediated signaling pathway, inositol trisphosphate biosynthetic process, G-protein coupled purinergic nucleotide receptor signaling pathway, phosphatidylinositol-mediated signaling,
GOTERM_CC_DIRECT	nucleoplasm, plasma membrane, integral component of plasma membrane, integral component of membrane,
GOTERM_MF_DIRECT	lipopolysaccharide binding, lipopolysaccharide receptor activity, platelet activating factor receptor activity, phospholipid binding, G-protein coupled purinergic nucleotide receptor activity,
INTERPRO	G protein-coupled receptor, rhodopsin-like, Platelet-activating factor receptor, GPCR, rhodopsin-like, 7TM,
KEGG_PATHWAY	Calcium signaling pathway, Neuroactive ligand-receptor interaction, Staphylococcus aureus infection,
PFAM	7 transmembrane receptor (rhodopsin family),
PUBMED_ID	11403499, 11438398, 11916258, 18021181, 19393038, 19565634,
REACTOME_PATHWAY	R-BTA-373076, R-BTA-416476,
UP_KEYWORDS	Cell membrane, Chemotaxis, Complete proteome, Disulfide bond, G-protein coupled receptor, Glycoprotein, Membrane, Pregnancy, Receptor, Reference proteome, Transducer, Transmembrane, Transmembrane helix,
UP_SEQ_FEATURE	chain:Platelet-activating factor receptor, disulfide bond, glycosylation site:N-linked (GlcNAc...), modified residue, topological domain:Cytoplasmic, topological domain:Extracellular, transmembrane region,
UP_TISSUE	Hypothalamus,
ENSBTAG00000014786	***polybromo 1(PBRM1)***
CHROMOSOME	22,
COG_ONTOLOGY	Chromatin structure and dynamics / Transcription,
ENSEMBL_GENE_ID	ENSBTAG00000014786,
GOTERM_BP_DIRECT	negative regulation of cell proliferation,
GOTERM_CC_DIRECT	nucleoplasm, BAF-type complex,
GOTERM_MF_DIRECT	chromatin binding,
INTERPRO	Bromo adjacent homology (BAH) domain, Bromodomain, High mobility group (HMG) box domain, Bromodomain, conserved site,
PFAM	Bromodomain, HMG (high mobility group) box, BAH domain,
PUBMED_ID	19393038,
REACTOME_PATHWAY	R-BTA-3214858,
SMART	BROMO, HMG, BAH,
UP_KEYWORDS	Bromodomain, Coiled coil, Complete proteome, Reference proteome,
UP_TISSUE	Uterus,
**Hip width (HW)**	
ENSBTAG00000015026	***ABL proto-oncogene 2, non-receptor tyrosine kinase(ABL2)***
CHROMOSOME	16,

EC_NUMBER	2.7.10.2,
ENSEMBL_GENE_ID	ENSBTAG00000015026,
GOTERM_BP_DIRECT	transmembrane receptor protein tyrosine kinase signaling pathway, positive regulation of cytosolic calcium ion concentration, regulation of autophagy, positive regulation of phospholipase C activity, positive regulation of neuron projection development, cell migration, actin cytoskeleton organization, regulation of endocytosis, cell differentiation, regulation of cell adhesion, peptidyl-tyrosine autophosphorylation, regulation of cell proliferation, innate immune response, positive regulation of oxidoreductase activity, cellular response to retinoic acid,
GOTERM_CC_DIRECT	extrinsic component of cytoplasmic side of plasma membrane,
GOTERM_MF_DIRECT	magnesium ion binding, non-membrane spanning protein tyrosine kinase activity, ATP binding, manganese ion binding,
INTERPRO	Protein kinase, catalytic domain, SH2 domain, Serine-threonine/tyrosine-protein kinase catalytic domain, Src homology-3 domain, Tyrosine-protein kinase, active site, Protein kinase-like domain, F-actin binding, Protein kinase, ATP binding site, Tyrosine-protein kinase, catalytic domain,
KEGG_PATHWAY	ErbB signaling pathway, Ras signaling pathway, Viral myocarditis,
PFAM	SH2 domain, SH3 domain, Protein tyrosine kinase, F-actin binding,
PUBMED_ID	19393038, 2081596,
REACTOME_PATHWAY	R-BTA-428890,
SMART	TyrKc, SH2, SH3, FABD,
UP_KEYWORDS	ATP-binding, Complete proteome, Kinase, Nucleotide-binding, Reference proteome, SH3 domain, Transferase, Tyrosine-protein kinase,
**Body length (BL)**	
100336611	***contactin associated protein like 5(CNTNAP5)***
ENTREZ_GENE_ID	100336611,
GOTERM_CC_DIRECT	integral component of membrane,
INTERPRO	Coagulation factor 5/8 C-terminal type domain, Epidermal growth factor-like domain, Laminin G domain, Fibrinogen, alpha/beta/gamma chain, C-terminal globular domain, Galactose-binding domain-like, Concanavalin A-like lectin/glucanase, subgroup,
PFAM	Laminin G domain,
PUBMED_ID	19393038,
SMART	EGF, LamG,
UP_KEYWORDS	Complete proteome, Disulfide bond, Membrane, Reference proteome, Repeat, Transmembrane, Transmembrane helix,
444863	***glycophorin C (Gerbich blood group)(GYPC)***
CHROMOSOME	2,
ENSEMBL_GENE_ID	ENSBTAG00000014863,
GOTERM_CC_DIRECT	integral component of membrane, cortical cytoskeleton,
GOTERM_MF_DIRECT	oligosaccharide binding,
INTERPRO	Neurexin/syndecan/glycophorin C,
KEGG_PATHWAY	Malaria,
PUBMED_ID	16912075, 19393038,
SMART	4.1m,

UP_KEYWORDS	Complete proteome, Membrane, Reference proteome, Transmembrane, Transmembrane helix,
UP_TISSUE	Heart ventrical,
100336680	***multiple EGF like domains 11(MEGF11)***
ENSEMBL_GENE_ID	ENSBTAG00000034140,
ENTREZ_GENE_ID	100336680,
PUBMED_ID	19393038,
525480	***solute carrier family 16 member 4(SLC16A4)***
CHROMOSOME	3,
ENSEMBL_GENE_ID	ENSBTAG00000015380,
GOTERM_BP_DIRECT	plasma membrane lactate transport, transmembrane transport,
GOTERM_CC_DIRECT	integral component of plasma membrane, integral component of membrane,
GOTERM_MF_DIRECT	lactate transmembrane transporter activity,
INTERPRO	Major facilitator superfamily, Major facilitator superfamily domain,
PFAM	Major Facilitator Superfamily,
PUBMED_ID	18996437, 19393038,
UP_KEYWORDS	Complete proteome, Membrane, Reference proteome, Transmembrane, Transmembrane helix,
UP_TISSUE	Kidney,
**Ear length (EL)**	
ENSBTAG00000012558	***ADAM metallopeptidase with thrombospondin type 1 motif 12(ADAMTS12)***
CHROMOSOME	20,
ENSEMBL_GENE_ID	ENSBTAG00000012558,
GOTERM_BP_DIRECT	cell-matrix adhesion, cell migration, proteoglycan catabolic process, negative regulation of chondrocyte differentiation, regulation of inflammatory response, proteolysis involved in cellular protein catabolic process, cellular response to interleukin-1, cellular response to tumor necrosis factor, cellular response to BMP stimulus, regulation of endothelial tube morphogenesis, negative regulation of hepatocyte growth factor receptor signaling pathway, negative regulation of cellular response to vascular endothelial growth factor stimulus, negative regulation of cellular response to hepatocyte growth factor stimulus,
GOTERM_CC_DIRECT	proteinaceous extracellular matrix,
GOTERM_MF_DIRECT	metalloendopeptidase activity, zinc ion binding,
INTERPRO	Thrombospondin, type 1 repeat, Peptidase M12B, ADAM/reprolysin, Peptidase M12B, propeptide, ADAM-TS Spacer 1, PLAC, Peptidase M12B, ADAM-TS, Metallopeptidase, catalytic domain,
PFAM	Thrombospondin type 1 domain, Reprolysin (M12B) family zinc metalloprotease , Reprolysin family propeptide, ADAM-TS Spacer 1,
SMART	TSP1,
UP_KEYWORDS	Complete proteome, Disulfide bond, Reference proteome, Signal,
ENSBTAG00000034140	***multiple EGF like domains 11(MEGF11)***
ENSEMBL_GENE_ID	ENSBTAG00000034140,
ENTREZ_GENE_ID	100336680,
